# Multi-omics reveals regulatory networks and critical early-warning factors for severe disease progression in diabetic patients infected with SARS-CoV-2

**DOI:** 10.3389/fimmu.2026.1871190

**Published:** 2026-09-18

**Authors:** Lei Dong, Dongshan Yu, Yunfeng Xiao, Jianjie Zhou, Jinhua Tang, Ying Li, Shijie Qin, Yueyun Ma, Yanhua Li

**Affiliations:** 1 Department of Clinical Laboratory, Air Force Medical Center, Air Force Medical University, Beijing, China; 2 Department of Infectious Diseases, The Second Affiliated Hospital of Nanchang University, Nanchang, China; 3 Department of Pharmacy, Hospital of the Shaanxi Provincial Corps of The Chinese People’s Armed Police Force, Xi´an, China; 4 Laboratory of Pathogen Microbiology and Immunology, Institute of Microbiology, Chinese Academy of Sciences, Beijing, China; 5 Innovative Vaccine and Immunotherapy Research Center, The Second Affiliated Hospital Zhejiang University School of Medicine, Hangzhou, China

**Keywords:** biomarkers, COVID-19, diabetes mellitus, disease progression, multi-omics, Omicron variant

## Abstract

**Background:**

Diabetic patients face elevated risks of severe COVID-19, yet the molecular underpinnings of disease progression, particularly for Omicron subvariants, which have predominated since late 2021, remain poorly defined. This observational study leverages a cohort recruited during China’s Omicron peak (December 2022–February 2023) to delineate multi-omic signatures underlying severe diabetic COVID-19.

**Methods:**

We enrolled 55 patients across five clinical strata: non-diabetic mild/severe, diabetic mild/severe/fatal. Integrated 4D-DIA proteomics and LC/GC-MS metabolomics were applied, adjusting for confounders while retaining secondary infections as integral disease features.

**Results:**

Diabetic patients exhibited immune exhaustion with elevated IL-6/IL-10, blunted antiviral responses, and high secondary infection rates. We identified 62 diabetes-unique molecules associated with coordinated dysregulation across six pathways: oxidative stress, ferroptosis, glycolytic dysfunction, lipid remodeling, insulin signaling, and endothelial injury. A graded molecular signature tracked clinical deterioration: progressive depletion of GP1BB and PRG3, coupled with stepwise elevation of MMP-3/LOXL1 and Dl-Xylose. Fatal cases further revealed a metabolic substrate misalignment, glycolytic flux adduct accumulation paradoxically coexisting with glucose, lactate, ornithine depletion, suggesting terminal fuel utilization failure. Stage-dependent shifts of stress mediators (e.g., GSK3B, ALDH9A1) distinguished severe from fatal outcomes, implying transition from compensatory adaptation to homeostatic exhaustion.

**Conclusions:**

Severe diabetic COVID-19 is characterized by progressive immune-metabolic collapse. GP1BB, MMP-3, and Dl-Xylose warrant evaluation as early-warning biomarkers, while ornithine and PC(18:2) may track homeostatic reserve depletion in terminal disease. Together, these findings identify candidate biomarkers and pathway nodes that, with further validation, could contribute to risk-stratification and therapeutic strategies for diabetic patients with severe viral infections.

## Graphical Abstract

**Figure:**
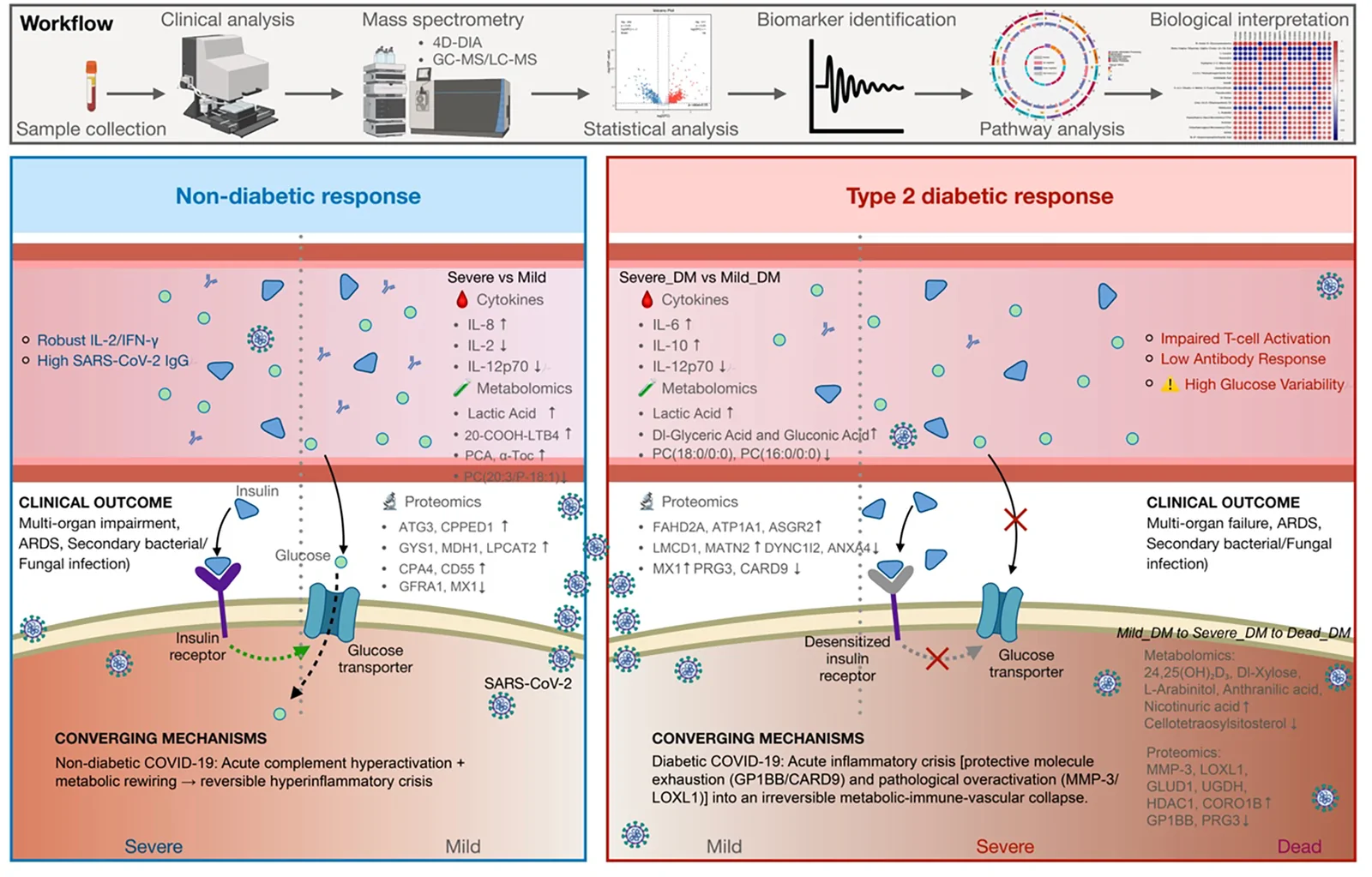

## Background

Diabetes is widely recognized as a critical comorbidity associated with an increased risk of severe outcomes in COVID-19 (1). Patients with diabetes, especially type 2 diabetes, exhibit a higher propensity for hospitalization, intensive care unit admission, and mortality, particularly when glycemic control is suboptimal (e.g., HbA1c ≥ 9%) or insulin therapy is required (2). This elevated vulnerability is thought to arise from multiple pathophysiological mechanisms, including dysregulated immune responses, chronic low-grade inflammation, and frequent coexisting conditions such as obesity and cardiovascular disease (3, 4).

Importantly, although multi-omics mechanisms governing virus-metabolism crosstalk during COVID-19 remain an emerging field, extensive epidemiological and mechanistic evidence from influenza research has firmly established that T2DM patients are inherently susceptible to severe respiratory viral infections, supporting a generalized diabetic vulnerability to viral stressors, large population-based cohorts have demonstrated that T2DM significantly increases influenza-related hospitalization, acute respiratory deterioration, and mortality, independent of traditional cardiovascular risk factors (5). Consistent with COVID-19, influenza infection also disrupts systemic glucose homeostasis, induces insulin resistance, and creates a bidirectional virus-metabolic vicious cycle, indicating that viral perturbation of metabolic immunity represents a shared pathogenic feature in T2DM viral complications (6). Despite growing clinical awareness, the precise biological pathways underlying this association remain incompletely elucidated.

A key unresolved question is why certain individuals with diabetes experience markedly severe COVID-19 outcomes, while others with similar glycemic profiles and comorbidities follow a milder clinical course. This heterogeneity suggests the influence of additional factors, such as variations in immune reactivity, genetic susceptibility, or undiagnosed microvascular and macrovascular complications (7). For example, studies indicate that SARS-CoV-2 can infect pancreatic β-cells via ACE2 receptors, potentially impairing insulin secretion and exacerbating hyperglycemia. This hyperglycemic state may activate signaling pathways such as NF-κB, increasing TNF-α production and impairing CD4+ T-cell function, ultimately leading to a condition resembling “immune paralysis” (8). Nobs SP et al. found hyperglycemia leads to impaired costimulatory molecule expression, antigen transport and T cell priming in distinct lung dendritic cell subsets, induced a defective antiviral adaptive immune response, delayed viral clearance and enhanced mortality, highlight a hyperglycemia-driven metabolic-immune axis (9). However, the specific glycemic thresholds that potentiate these adverse outcomes have not been clearly established (10).

Another significant area of uncertainty is the extent to which glycemic control influences COVID-19 severity in diabetic patients. Although maintaining good glycemic control is broadly recommended, its direct role in mitigating severe outcomes remains debated. Some studies suggest that improved glucose management may lower complication risks, whereas others report no significant association between pre-infection HbA1c levels and COVID-19 severity (11, 12). These conflicting findings raise questions about the potential benefit of glycemic interventions specifically aimed at reducing mortality or hospitalization in this population.

Moreover, the serological and molecular profiles, including characteristics of immune response, proteomic signatures, and metabolomic alterations, in diabetic patients with COVID-19 are not well characterized. Diabetes is associated with chronic inflammation and insulin resistance, which may contribute to disease progression (13). However, the exact mechanisms linking these conditions to severe illness or fatality have yet to be fully delineated. Hyperglycemia, for instance, may facilitate viral replication or disrupt immune cell function, but the clinical significance of these effects in real-world settings remains unclear (14). Addressing these knowledge gaps is essential for developing targeted strategies to improve outcomes in this high-risk population during and beyond the pandemic.

In light of these unresolved issues, this study aims to clarify the biological pathways connecting diabetes to severe COVID-19, identify factors associated with high risk, and establish a scientific foundation for therapeutic strategies that may reduce morbidity and mortality in this vulnerable group.

## Methods

### General information and diagnostic criteria

This study enrolled 55 patients from the Air Force Medical Center (December 2022–February 2023), with approval from the institutional ethics committee (Approval No. 2023-30-PJ01). All participants were classified according to established clinical guidelines. Type 2 diabetes mellitus (T2DM) was diagnosed per the Chinese Diabetes Prevention and Control Guidelines (2023 edition) (15), requiring typical symptoms plus one of the following: random blood glucose ≥11.1 mmol/L, fasting blood glucose ≥7.0 mmol/L, 2-hour OGTT glucose ≥11.1 mmol/L, or HbA1c ≥6.5%. COVID-19 diagnosis followed the Diagnosis and Treatment Protocol for COVID-19 (Trial Version 10) (16), based on clinical manifestations plus a positive SARS-CoV-2 nucleic acid/antigen test, viral culture, or a ≥4-fold rise in SARS-CoV-2-specific IgG during convalescence. Severe COVID-19 in adults was defined as meeting any of: respiratory rate ≥30 breaths/min, SpO_2_ ≤93% on room air, PaO_2_/FiO_2_ ≤300 mmHg, or >50% lesion progression on lung imaging within 24–48 hours. Critical cases required mechanical ventilation, shock, or multi-organ failure necessitating ICU care (16). Patients were stratified into five groups: Mild (n=20), Severe (n=10), Mild_DM (n=15), Severe_DM (n=10), and deceased Severe_DM patients (Dead_DM, n=5). The study design is summarized in **Figure 1A**. Retrospective data (**Table 1**) indicated that the Severe, Severe_DM, and Dead_DM groups were generally older, predominantly male, and had more comorbidities (e.g., hypertension, hyperlipidemia). Vaccination rates were higher in the Mild and Mild_DM groups.

**Figure 1:**
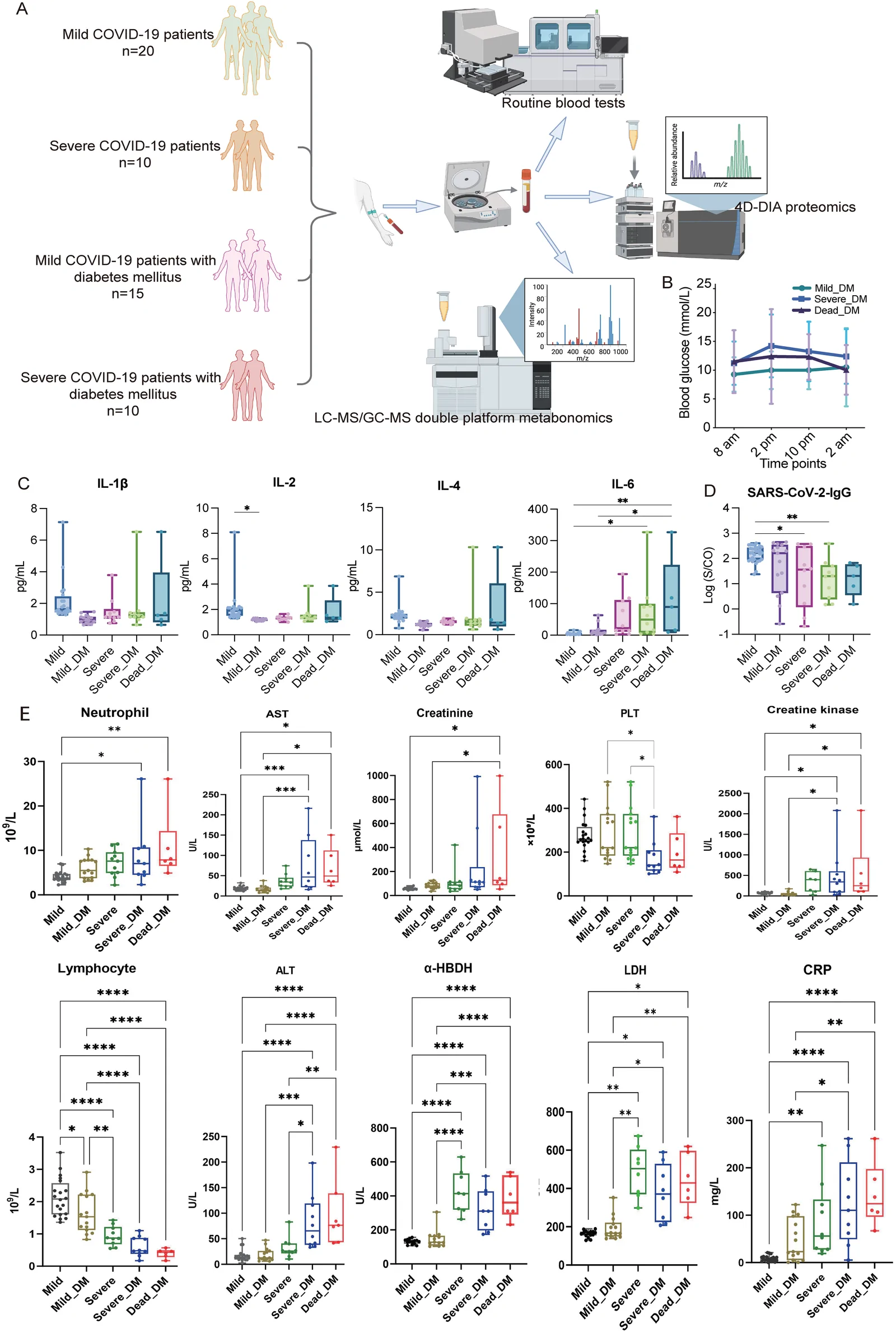
Clinical, glycemic, and immunological profiling of SARS-CoV-2-infected patients with or without type 2 diabetes (T2DM). **(A)** Study design and participant stratification. A total of 60 hospitalized patients with confirmed SARS-CoV-2 infection were stratified into five groups based on disease severity and T2DM comorbidity: mild non-diabetic (Mild, n = 20), severe non-diabetic (Severe, n = 10), mild diabetic (Mild_DM, n = 15), severe diabetic (Severe_DM, n = 10), and fatal diabetic (Dead_DM, n = 5). Serum samples collected within 24 h of admission were subjected to proteomic and metabolomic profiling. Created in BioRender. Ma, S. (2026) **(B)** 24-hour blood glucose variability across diabetic subgroups. Shaded bands represent interquartile ranges of continuous glucose monitoring; the dashed horizontal line denotes the hypoglycemia threshold (< 3.9 mmol/L). Hypoglycemic episodes occurred in 0/15 Mild_DM, 3/10 Severe_DM, and 5/5 Dead_DM patients. **(C)** Box plots illustrating the plasma levels of key inflammatory cytokines (IL-1β, IL-2, IL-4, IL-6, pg/mL) in different patient subgroups. **(D)** Box plot showing the log-transformed signal cut-off (S/CO) values of SARS-CoV-2 spike 2 IgG antibody (SARS-CoV-2-IgG) in peripheral blood. **(E)** Box plots summarizing the comparison of routine laboratory biomarkers, including immune cell counts (neutrophils, lymphocytes), liver injury markers (AST, ALT), myocardial and tissue damage indicators (α-HBDH, LDH, creatine kinase), renal function index (creatinine), platelet count (PLT), and systemic inflammation marker (CRP). Statistical significance was annotated as follows: *p < 0.05, **p < 0.01, ***p < 0.001, ****p < 0.0001. Mild, non-diabetic mild COVID-19 patients; Mild_DM, mild COVID-19 patients with diabetes mellitus; Severe_DM, surviving severe COVID-19 patients complicated with diabetes mellitus; Dead_DM, deceased severe COVID-19 patients with diabetes mellitus. T2DM, type 2 diabetes mellitus; DM, diabetes mellitus; CRP, C-reactive protein; PCT, procalcitonin; LDH, lactate dehydrogenase; CK, creatine kinase; α-HBDH, α-hydroxybutyrate dehydrogenase.

**Table 1:** General information of the participants.

Groups	Mild COVID-19 patients (mild)	Severe COVID-19 patients (severe)	Mild COVID-19 patients with diabetes mellitus (Mild_DM)	Severe COVID-19 patients with diabetes mellitus (Severe_DM)	Dead from severe COVID-19 with diabetes mellitus (Dead_DM)	P value
N (Male%)	20 (15.0%)	10 (80.0%)	15 (86.7%)	10 (90.0%)	5(100%)	N/A
Age, Year	39 (28, 45)	80 (69, 85)	63 (57, 72)	77 (61, 89)	87 (65, 90)	0.001
COVID-19 vaccination (%)	100%	20%	53.3%	20%	40%	N/A
Pre-existing medical condition, n						
Hypertension	N/A	7	8	7	5	N/A
Hyperlipidemia	N/A	N/A	4	2	4	N/A
Fatty liver	N/A	N/A	2	1	4	N/A
Lung cancer	N/A	N/A	1	N/A	N/A	N/A
Bronchial asthma	N/A	N/A	1	N/A	N/A	N/A
Kidney cysts	N/A	N/A	3	N/A	N/A	N/A
Arteriosclerosis	N/A	N/A	2	N/A	N/A	N/A
Cerebral infarction	N/A	3	3	2	1	N/A
Coronary heart disease	N/A	1	3	5	3	N/A
Pulmonary nodules	N/A	N/A	1	1	N/A	N/A
COPD	N/A	1	N/A	N/A	N/A	N/A
Renal hamartoma	N/A	N/A	N/A	N/A	N/A	N/A
Renal insufficiency	N/A	1	2	2	3	N/A
Senile dementia	N/A	1	N/A	N/A	N/A	N/A
Hypothyroidism	N/A	N/A	N/A	1	N/A	N/A
Sequelae of cerebral hemorrhage	N/A	N/A	N/A	1	1	N/A
Myocardial infarction	N/A	1	N/A	N/A	1	N/A
Chronic atrophic gastritis	N/A	1	N/A	N/A	N/A	N/A
Chronic gastritis	N/A	1	N/A	N/A	N/A	N/A
Diffuse large B lymphoma	N/A	N/A	N/A	1	N/A	N/A
Routine blood tests						
RBC, ×10^12^/L	4.50 (4.18, 4.77)	3.90 (3.52, 4.40)	3.88(3.22, 4.69)	4.25 (3.71, 4.56)	3.84(3.26, 4.62)	0.322
HGB, g/L	134.0 (129.0, 142.0)	124 (109, 134)	113.0 (99.0,136.0)	126.0 (105.0, 139.0)	117.0 (98.5,142.0)	0.052
**PLT**, ×10^9^/L	259.0 (246.0, 319.0)	202.0(124.0, 275.0)	220.0(134.0, 374.0)	140.5 (115.0, 208.5)	138.0 (122.0, 276.0)	**0.005**
Leukocyte Count, ×10^9^/L	6.62 (5.93, 7.74)	8.57 (6.43, 11.80)	7.21 (6.31, 10.7)	7.58 (5.35, 11.57)	8.82 (6.65, 19.42)	0.232
**Neutrophil**	3.80 (3.35, 4.79)	7.86 (4.75, 9.96)	5.48 (3.74,7.48)	6.10(4.57, 10.55)	7.60 (5.97, 18.27)	**0.001**
**Lymphocyte**	2.08 (1.60, 2.57)	0.87(0.68,1.21)	1.53 (1.12, 2.22)	0.48(0.38, 0.87)	0.40 (0.25, 0.47)	**0.001**
Monocyte	0.37 (0.32, 0.45)	0.36 (0.13, 0.69)	0.54 (0.43, 0.82)	0.39 (0.31, 0.89)	0.38 (0.27, 0.11)	0.103
**CRP**	20.0(15.6, 49.8)	27.8(18.9, 133.2)	21.30 (3.93, 21.08)	110.9(49.3, 211.5)	157.1(117.4, 240.2)	**0.005**
**PCT**	0.16(0.11, 0.47)	0.17(0.05, 0.97)	0.15(0.11, 0.65)	1.57 (0.13, 8.74)	2.10 (0.57, 6.75)	**0.029**
Ferritin	65.0 (34.5, 89.9)	437.0 (366.0, 667.1)	352.0 (216.75, 544.75)	806.6 (361.6, 1030.7)	832.2 (363.4, 1132.2)	0. 07
Fibrinogen	5.0 (3.60, 6.10)	4.35 (3.23, 5.46)	5.31 (3.65, 5.70)	4.28 (3.89, 5.88)	5.29 (4.11, 7.21)	0.475
**Glycated albumin**	12.5 (11.0, 15.0)	17.52 (15.95, 18.93)	26.1 (21.7, 30.5)	21.20 (20.50, 26.44)	22.73 (20.72, 28.98)	**0.008**
Triglycerides	1.06 (0.82, 1.57)	1.08 (0.86, 1.20)	1.36 (0.85,1.66)	1.04 (0.98, 1.70)	1.02 (0.88, 1.99)	0.093
Hs-TnT	0.20 (0.04, 3.60)	0.10 (0.025, 4.24)	2.34 (0.20, 4.50)	17.50 (3.38, 61.90)	25.43 (13.38, 79.90)	0.275
**LDH**	166.0 (154.0, 174.0)	503.0 (369.4, 603.3)	166.50 (149.25, 221.50)	370.9 (224.0, 528.9)	392.2 (299.0, 527.9)	**0.001**
**Creatine kinase**	79.0 (63.0, 87.0)	404.5 (118.3,586.7)	32.5 (24.5, 68.5)	206.5(61.5,700.7)	111.0 (71.0, 1191.0)	**0.001**
CK-MB	1.20 (.030, 2.16)	2.78(1.16, 5.48)	1.64 (1.04, 2.38)	1.15 (0.48, 2.15)	1.10 (0.65, 3.90)	0.232
**α-HBDH**	131.5(118.3, 141.0)	413.5 (319.0, 532.0)	127.5 (107.8, 165.5)	310.5 (197.3,426.8)	311.0 (270.5, 464.0)	**0.001**
TC	5.25 (4.27, 5.74)	4.13 (3.35, 4.90)	3.67 (3.28, 4.68)	3.04(2.80, 3.74)	2.98 (2.71, 3.98)	0.060
Liver function						
**ALT, U/L**	13.5 (9.2,18.4)	25.9(20.0, 40.6)	12.7(9.3, 26.2)	40.0 (20.8, 89.5)	41.5(18.4, 102.7)	**0.008**
**AST, U/L**	19.8 (15.1,22.4)	34.5(23.6,44.9)	14.7(12.0, 28.7)	38.6 (19.7, 112.2)	37.1 (13.6, 124.9)	**0.001**
**TBil, μmol/L**	12.1(10.1, 17.6)	13.5 (7.1, 39.8)	9.0 (5.0, 11.2)	28.5 (11.8,79.4)	24.3 (15.6,67.5)	**0.025**
**Albumin, g/L**	46.3 (45.0, 47.2)	33.5 (30.2, 36.8)	36.6 (34.7, 41.6)	33.3 (31.4, 38.9)	30.3 (28.1, 36.4)	**0.010**
**Creatinine, μmol/L**	56.4(52.7, 65.8)	87.0 (54.0, 110.4)	79.7 (64.1, 103.9)	112.3 (70.7, 237.0)	129.3 (104.0, 776.0)	**0.001**

COPD, Chronic obstructive pulmonary disease; RBC, Red blood cell; HGB, Hemoglobin;PLT, Platelets; Hs-TnT, High-sensitivity cardiac troponin T; CRP, C-reactive protein; PCT, Procalcitonin; LDH, Lactic dehydrogenase; CK-MB, Creatine kinase-MB; α-HBDH, α-hydroxybutyrate dehydrogenase; TC, Total Cholesterol; ALT, Alanine Aminotransferase; AST, Aspartate Aminotransferase; TBil, Total bilirubin.

Bold values indicate statistically significant differences between groups (p < 0.05).

### Clinical and serological analyses

Routine clinical indicators were performed at the Department of Clinical Laboratory of the Air Force Medical Center, China. Blood glucose (GLU) level was frequently measured for patients with DM. FACSLyric™ flow cytometer (BD Biosciences, USA) were used to detect cytokines including interferon (IFN)-γ, interleukin (IL)-1β, IL-2, IL-4, IL-5, IL-6, IL-8, IL-10, IL-12p70, IL17A, IL17F, IL-22, tumor necrosis factor (TNF)-α, and TNF-β with the protocol of the kit manual. First, the serum samples and standards were prepared. 20 μL serum sample, 10 μL detection antibody, 10 μL capture microspheres and 20 μL diluent were added to each sample tube, followed with mixture and incubation at room temperature without light. Then 20 μL PE-labeled streptavidin was added, mixed and incubated at room temperature without light. After washing twice, the sample resuspended with 150 μL 1 × buffer. Subsequently, the fluorescence corresponding to each cytokine was detected on the flow cytometer. Finally, the standard curve of the twelve standards of cytokines was combined to achieve quantitative results.

SARS-CoV-2 IgG was detected using a magnetic particle chemiluminescence kit (Autobio, Cat. 30041613CM) targeting the ancestral spike protein (SP). In brief, serum was applied to the magnetic particles which had been coated with the SP. Then the enzyme conjugate was prepared through anti-human IgG antibody labeled with horseradish peroxidase (HRP), leading to a solid-phase antigen-antibody enzyme-labeled secondary antibody complex. Finally, the intensity of photons released from the luminescent substrate catalyzed by the complexes was proportional to the content of IgG-type antibodies to SARS-CoV-2 SP.

### Proteomic profiling

Serum samples were retrieved from −80 °C storage and thawed on ice. High-abundance proteins were depleted using the EasyPept Deep Depletion Kit (Yisuan Biotechnology, China) according to the manufacturer’s protocol. Briefly, 100 µL of serum was incubated with 1 mg of magnetic nanomaterial suspension at 37 °C for 1 h with gentle rotation. After magnetic separation and washing with Wash Buffer (three times), the captured proteins were subjected to reduction, alkylation, and enzymatic digestion. The protein-bound beads were resuspended in 40 µL of Reagent A (reduction/alkylation and digestion buffer), followed by the addition of 1.2 µL of Reagent B and incubation at 95 °C for 5 min (1000 rpm). After cooling to room temperature, 2 µL of Reagent C and 5 µL of Reagent D (trypsin) were added, and the mixture was incubated at 37 °C for 2 h. The digestion was terminated by adding 3 µL of Reagent E, and the supernatant was collected after centrifugation at 20,000×g for 1 min. The resulting peptide mixtures were desalted using C18 tips. Briefly, the tips were activated with 100 µL of methanol, conditioned with 100 µL of Condition buffer (70% ACN, 0.2% TFA), and equilibrated with 100 µL of Wash buffer (0.2% TFA). The peptide samples (1–30 µg) were loaded onto the tips, washed twice with 100 µL of Wash buffer, and eluted twice with 30 µL of Elution buffer (90% ACN, 0.2% TFA). The eluates were pooled and concentrated using a vacuum centrifugal concentrator. Peptide concentrations were determined using a Nanodrop spectrophotometer.

A pooled sample containing equal amounts of peptide digests from all specimens was used to generate the spectral library via data-dependent acquisition (DDA) on the same chromatographic and mass spectrometric setup. For liquid chromatography, peptide separation was performed on a C18 analytical column (25 cm × 75 µm ID, 1.6 µm particle size, Ionopticks) using a nanoElute system (Bruker) at a flow rate of 300 nL/min. The mobile phases consisted of buffer A (0.1% formic acid in water) and buffer B (0.1% formic acid in acetonitrile). The gradient was programmed as follows: 0 min, 2% B; 45 min, 22% B; 50 min, 37% B; 55 min, 80% B; 60 min, 80% B. Mass spectrometry analysis was performed on a timsTOF Pro2 instrument (Bruker) with a CaptiveSpray source. The DIA method parameters were: capillary voltage, 1.4 kV; dry temperature, 180 °C; dry gas, 3.0 L/min; mass range, 100–1700 m/z; ion mobility range, 0.7–1.3 V·s/cm²; and collision energy, 20–59 eV.

DIA data were analyzed directly using Spectronaut Pulsar 17.5 (Biognosys, Switzerland) in directDIA mode, without the need for a separate DDA spectral library. The search was performed against the UniProt human database (uniprot-Homo sapiens-9606-2023.2.1.fasta) with the following parameters: trypsin/P as enzyme, maximum missed cleavages = 2, carbamidomethylation of cysteine as fixed modification, and oxidation of methionine and acetylation of protein N-terminus as variable modifications. The precursor and protein Q-value cutoffs were set at 0.01, and quantification was performed at the MS2 level. For differential abundance analysis, data were normalized using the median normalization algorithm in Spectronaut. Proteins with ≥1 unique peptide and with valid values in at least 50% of samples within at least one group were retained; missing values within groups were imputed using the group mean, and remaining missing values were replaced with half of the minimum value. Data were then median-normalized and log2-transformed. Proteins with fold change > |1.2|, p-value < 0.05 were considered differentially expressed proteins (DEPs). All identified proteins were annotated using Gene Ontology (http://www.blast2go.com/b2ghome; http://geneontology.org/) and Kyoto Encyclopedia of Genes and Genomes (KEGG) pathway databases (http://www.genome.jp/kegg/). DEPs were subjected to GO and KEGG enrichment analyses. Protein-protein interaction networks were constructed using the STRING database (https://string-db.org/).

### LC-MS/GC-MS metabolomic analysis

We employed liquid chromatography-tandem mass spectrometry (LC-MS/MS) and gas chromatography-mass spectrometry (GC-MS) for comprehensive metabolomic profiling. Serum samples were thawed on ice, and 150 µL of each sample was transferred to a 1.5 mL Eppendorf tube. Protein precipitation was performed by adding 600 µL of ice-cold methanol/acetonitrile (2:1, v/v) containing mixed internal standards (L-2-chlorophenylalanine, succinic acid-d4, L-valine-d8, and cholic acid-D4; each at 4 µg/mL). The mixture was vortexed for 1 min, sonicated in an ice-water bath for 10 min, and incubated at −40 °C overnight. After centrifugation at 12,000 rpm for 10 min at 4 °C, 150 µL of the supernatant was filtered through a 0.22-µm organic-phase syringe filter for LC-MS/MS analysis. For GC-MS analysis, another 150 µL aliquot of the supernatant was transferred to a glass derivatization vial and dried under a centrifugal concentrator. The dried residue was subjected to oximation with 80 µL of methoxyamine hydrochloride in pyridine (15 mg/mL) at 37 °C for 60 min, followed by derivatization with 50 µL of BSTFA and 20 µL of n-hexane, along with 10 µL of a mixture of fatty acid methyl ester internal standards (C8, C9, C10, C12, C14, C16, C18, C20, C22, C24, each dissolved in chloroform), at 70 °C for 60 min. After cooling to room temperature for 30 min, the samples were analyzed by GC-MS. Quality control (QC) samples were prepared by pooling equal volumes of extracts from all samples.

LC-MS/MS analysis was conducted using a Waters ACQUITY UPLC I-Class Plus system coupled to a Thermo Q-Exactive mass spectrometer. Chromatographic separation was performed on an ACQUITY UPLC HSS T3 column (100 mm × 2.1 mm, 1.8 µm, Waters) maintained at 45 °C. Mobile phases consisted of (A) water with 0.1% formic acid and (B) acetonitrile. The gradient elution at a flow rate of 0.35 mL/min was as follows: 0 min, 5% B; 2 min, 5% B; 4 min, 30% B; 8 min, 50% B; 10 min, 80% B; 14 min, 100% B; 15 min, 100% B; 15.1 min, 5% B; and 16 min, 5% B. The injection volume was 3 µL. Mass spectrometry was performed in both positive and negative ionization modes with the following parameters: spray voltage, 3800 V (positive) and −3000 V (negative); capillary temperature, 320 °C; auxiliary gas heater temperature, 350 °C; sheath gas flow rate, 35 Arb; auxiliary gas flow rate, 8 Arb; S-lens RF level, 50; mass range, m/z 100–1200; full MS resolution, 70,000; MS/MS resolution, 17,500; and normalized collision energy (NCE) stepped at 10, 20, and 40 eV.

GC-MS analysis was performed on an Agilent 7890B-5977A system equipped with a DB-5MS capillary column (30 m × 0.25 mm × 0.25 µm, Agilent J&W Scientific). Helium (≥99.999%) was used as carrier gas at a flow rate of 1.0 mL/min. The injector temperature was set at 260 °C, and 1 µL of sample was injected in splitless mode. The column oven temperature program was: initial 60 °C held for 0.5 min, ramped to 125 °C at 8 °C/min, to 210 °C at 8 °C/min, to 270 °C at 15 °C/min, and finally to 305 °C at 20 °C/min, held for 5 min. The electron impact (EI) ion source was operated at 70 eV, with the ion source temperature at 230 °C and the quadrupole temperature at 150 °C. Full-scan mode was employed with a mass range of m/z 50–500.

Data processing and identification. For LC-MS/MS data, raw spectral data were processed using Progenesis QI v3.0 (Nonlinear Dynamics, Newcastle, UK) for baseline filtering, peak detection, integration, retention time correction, peak alignment, and normalization. Metabolite identification was performed by matching accurate mass, retention time, and MS/MS fragmentation patterns against the Human Metabolome Database (HMDB), Lipidmaps (v2.3), METLIN, and the in-house LuMet-Animal 3.0 database. Precursor tolerance was set at 5 ppm for HMDB and Lipidmaps, and 10 ppm for LuMet-Animal and METLIN; product tolerance was 10 ppm for HMDB and Lipidmaps, and 20 ppm for LuMet-Animal and METLIN. Identification confidence levels were assigned as follows: Level 1, retention time ±0.3 min with fragmentation score ≥45; Level 2, retention time ±0.3 min with fragmentation score <45; Level 3, fragmentation score ≥45; Level 4, fragmentation score <45. Only metabolites with a score ≥36 (out of 80) were retained.

For GC-MS data, raw files (.D format) were converted to.abf format using AnalysisBaseFileConverter and processed using MS-DIAL v4.24 for peak detection, deconvolution, identification, alignment, and filtering. Metabolite identification was performed using the in-house LuMet-GC 5.0 database (containing 2,543 metabolites), with a total spectrum similarity score ≥70 (out of 100) for retention. Data were normalized using total peak area normalization (sample peak area/total peak area × mean total peak area of all samples) and internal standard-segmented normalization (sample peak area/internal standard peak area × mean internal standard peak area across all samples) for selected internal standards with RSD < 0.1.

Multivariate statistical analyses, including unsupervised principal component analysis (PCA) and supervised models (partial least squares discriminant analysis, PLS-DA; and orthogonal partial least squares discriminant analysis, OPLS-DA), were applied to distinguish metabolite profiles between groups. The robustness of PLS-DA and OPLS-DA models was evaluated by 200-fold permutation testing, with model performance assessed using R² and Q² values; overfitting was considered unlikely when the Q² intercept was <0 in permutation tests. Differential metabolites were selected based on variable importance in projection (VIP) values >1.0 and p < 0.05. Metabolite annotation was performed using KEGG and Reactome databases (https://reactome.org/).

### Combined proteomics and metabolomic analysis

Top 20 differentially expressed proteins and metabolites with the largest differences were selected (sorted by P-value, including all differentially expressed proteins or metabolites when the number of differentially expressed proteins or metabolites is less than 20), and Pearson correlation was used to analyze the correlation between proteomics and metabolomic based on the relative content data. Based on Pearson correlation analysis, the correlation between protein and metabolite response intensity data was analyzed, and the relationship with p-value <0.05 and correlation >0.95 was selected to draw the network diagram. KGML network analysis was used to study the interactions between proteomics and metabolome.

### Statistical analysis

Clinical data conforming to normal distribution are presented as mean ± standard deviation ( $\bar{\text{x}}$ ± s) and compared using t-test or one-way ANOVA. Skewed data are presented as median (interquartile range, IQR) and analyzed using Mann-Whitney U or Kruskal-Wallis H tests. Statistical analysis was performed using GraphPad Prism (v9.4.3). All p-values were two-tailed, with p<0.05 considered statistically significant. Raw normalized abundance data of proteome and metabolome profiles were corrected for clinical confounding factors using multiple linear regression respectively. Six clinical covariates, including age, BMI, sex, comorbidities, vaccination doses and vaccine type, secondary infection was excluded because we considered it as an integral factor of the disease severity, were incorporated into the regression model. For each protein and metabolite, original quantitative value was taken as the dependent variable, while the above clinical indicators were set as independent variables. Residual values derived from regression fitting were adopted as corrected molecular abundance matrix for all subsequent downstream analyses. Multicollinearity among covariates was assessed via the distribution of Spearman correlation coefficients. Box plots were applied to quantify the contribution of each confounding factor to total molecular variation of proteome and metabolome datasets, verifying the stability of correction model and ensuring that genuine biological variation was largely preserved in corrected residuals after noise removal. All regression calculation and data visualization were performed in R software. Normally distributed quantitative data of metabolites underwent t-tests or one-way ANOVA for intergroup comparisons. Differential metabolites were selected based on variable importance in projection (VIP) values >1.0 and statistical significance (p < 0.05).

## Results

### Clinical features showed the blood microenvironment in patients with diabetes mellitus is more severely disordered following SARS-CoV-2 infection than in non-diabetic patients.

The study design was summarized in **Figure 1A**. Analysis of clinical characteristics and laboratory parameters revealed several key findings (1): Platelet counts were significantly lower in the Severe_DM and Dead_DM groups (p=0.005) (2). Neutrophil counts increased progressively from the Mild_DM and Mild groups to the Severe, Severe_DM, and Dead_DM groups (p = 0.001), indicating progressive complicated bacterial infection. 0/20 in Mild group, 8/15 in Mild_DM group were progressed to secondary bacterial infection, and most infections were caused by a single or two bacteria. Whereas, 7/10 in Severe group, 9/10 in Severe_DM group and 5/5 in Dead_DM group developed secondary bacterial infection, and all secondary infections in the DM groups were polymicrobial infections. Three cases in the Dead_DM group presented with secondary fungal infection, indicating complicated secondary infections were associated with poor prognosis. RBC and WBC counts showed no significant differences (3). Lymphocyte counts were comparable among Severe, Severe_DM, and Dead_DM groups but were significantly lower than those in the Mild and Mild_DM groups (p=0.001) (4). CRP and PCT were markedly elevated in the Severe_DM and Dead_DM groups (p=0.005 and p =0.029, respectively) (5). Glycated albumin levels were higher in all diabetic groups, consistent with diabetes, though no significant differences were observed among the Severe_DM, Dead_DM, and Mild_DM groups (6). Muscle enzyme profiles (LDH, creatine kinase, α-HBDH) were significantly elevated in the Severe, Severe_DM, and Dead_DM groups (p=0.001) (7). Compared to the Mild and Mild_DM groups, the Severe, Severe_DM, and Dead_DM groups exhibited increased ALT, AST, TBil, and Cr levels, alongside decreased albumin (**Table 1**, **Figure 1**). Collectively, these findings collectively indicate pronounced inflammation, myocardial injury, hepatic dysfunction, renal impairment and possible bacterial co-infection in severe cases.

Building upon these clinical observations, we next sought to determine whether glycemic instability contributed to this exacerbated phenotype. Blood glucose variability was further found significantly correlated with disease severity (**Figure 1B**). Mild diabetic patients displayed smaller, more stable glucose fluctuations, whereas severe and fatal diabetic groups showed greater variability and increased frequency of hypoglycemia (blood glucose <3.9 mmol/L: Severe = 3 cases, Dead = 5 cases, Mild = 0 case), reflecting disrupted glucose metabolism. Although statistical analysis indicated P > 0.05 for glucose levels across groups at different time points, potentially influenced by confounding factors such as diet, intravenous fluids, and hypoglycemic medications, the pronounced instability in severe and fatal cases underscores its association with adverse outcomes.

To understand the immunological underpinnings of these observations, we profiled circulating cytokines. Inflammatory cytokines including IL-1β, IL-2, IL-4 were elevated in non-diabetic mild COVID-19 patients compared to diabetic mild and severe groups, suggesting an early and effective antiviral immune response in non-diabetics. IL-2, critical for T-cell activation, was notably higher in non-diabetic mild cases, indicating robust adaptive immunity. In contrast, IL-6, a central mediator of cytokine storm, was significantly elevated in diabetic severe and fatal groups, highlighting a tendency toward inflammatory dysregulation. Additionally, SARS-CoV-2-specific antibody levels were significantly higher in the non-diabetic mild group compared to all diabetic and non-diabetic severe groups (**Figure 1D**). These results collectively indicate that non-diabetic patients mount a more effective early immune response, facilitating timely viral clearance, whereas diabetic patients display compromised protective immunity, leading to delayed clearance and dysregulated inflammation.

### 4D-DIA proteomic characteristics of COVID-19 patient with and without T2DM

Next, we transitioned to a high-resolution proteomic analysis to uncover the molecular machinery driving these phenotypes. We adjusted for potential clinical confounders (age, BMI, sex, comorbidities, vaccine type, and dose number) in the proteomic analysis. Spearman rank correlation analysis showed all covariates had correlation coefficients clustered within the −0.5 to 0.5 range, with no strong inter-variable correlations or multicollinearity. Boxplot quantification of variance contributions from each confounder revealed uniformly low median values across all factors, with only sporadic mild individual-level interference detected in a small subset of samples. Residuals after adjustment accounted for the vast majority of total data variance, indicating these confounders introduced only weak, non-systemic bias that did not mask true biological signals (**Supplementary Figure 1**). Adjustment thus improved the signal-to-noise ratio without attenuating target study effects. Proteomic profiling of serum samples identified 2,025 high-confidence proteins. Principal component analysis (PCA) based on protein expression clearly distinguished between compared groups (**Figure 2A**), indicating that the protein profiles of patients in the compared groups have different characteristics. Venn and pairwise comparisons further confirmed distinct protein expression patterns with minimal overlap (**Figure 2B**; **Supplementary Figures 1E, F**; **Supplementary Tables 1**-**S5**), underscoring significant differential expression between conditions. Comparative analysis between non-diabetic severe and mild groups revealed 344 significantly dysregulated proteins, including 290 upregulated (e.g., CPPED1, ATG3, TRA2A, CPA4, DCAF7) and 54 downregulated (e.g., GFRA1, GLTPD2, MMP14, FASTKD5, MX1) (**Figures 2B, C**; **Supplementary Figure 1**; **Supplementary Table 2**). This pattern suggests a heightened stress state in severe patients, marked by amplified immune/inflammatory responses and suppressed repair/metabolic processes, potentially increasing risk of excessive inflammation, cell death, and organ injury.

**Figure 2:**
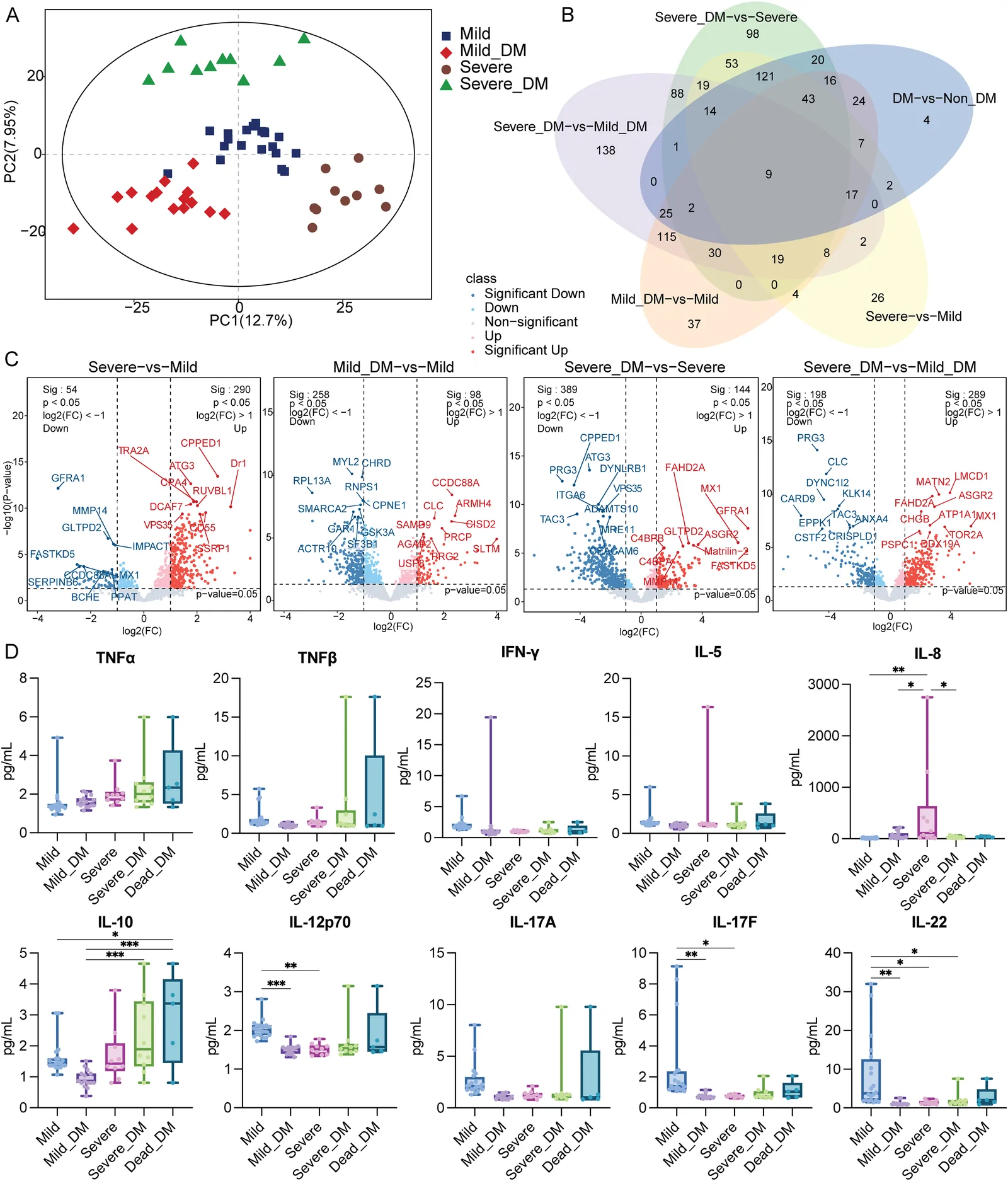
Quantitative proteomic profiling reveals distinct molecular signatures across disease severity and diabetic status. **(A)** Principal component analysis (PCA) of the global proteomic landscape. **(B)** Venn diagrams illustrating the overlap of DEPs among comparisons. **(C)** Volcano plots depicting differentially expressed proteins (DEPs) across four key comparisons. Red dots denote significantly upregulated proteins, while blue dots denote significantly downregulated proteins (FDR < 0.05, |log_2_FC| ≥ 1). The number of DEPs for each comparison is indicated: Severe vs. Mild (290 up, 54 down), Mild_DM vs. Mild (198 up, 112 down), Severe_DM vs. Severe (234 up, 175 down), and Severe_DM vs. Mild_DM (289 up, 198 down). **(D)** Targeted validation of key inflammatory proteins by FCM. Protein levels of IL-17F, IL-22, IL-12p70, IL-8, and IL-10 were quantified in serum. Statistical significance was determined by one-way ANOVA with Tukey’s *post-hoc* test. Data are presented as mean ± SEM. *p < 0.05, **p < 0.01, ***p < 0.001, ns, not significant. DEPs, differentially expressed proteins; FC, fold change; FDR, false discovery rate; T2DM, type 2 diabetes mellitus; PCA, principal component analysis; FCM, flow cytometry.

In diabetic severe versus mild COVID-19 patients, we identified 289 upregulated and 198 downregulated proteins (**Figures 2B, C**; **Supplementary Figure 1**; **Supplementary Table 5**). Key upregulated proteins in severe diabetic cases included LMCD1, MATN2, FAHD2A, ASGR2, and CHGB, while downregulated proteins featured PRG3, CLC, CARD9, and others. Notably, several proteins, such as CAPG, USP8, PSMA7, and PTGDS, etc., were uniquely changed in severe diabetic infection, absent in non-diabetic comparisons (**Figure 2C**; **Supplementary Figure 1**; **Supplementary Table 1**), indicating their involvement in diabetes-specific pathological mechanisms including hyperinflammation, metabolic reprogramming, insulin resistance, and increased opportunity infection.

Serum validation of cytokines corroborated these proteomic shifts. Inflammatory cytokines including IL-17F, IL-22, and IL-12p70 were elevated in non-diabetic mild COVID-19 patients compared to diabetic mild and severe groups, suggesting an early and effective antiviral immune response in non-diabetics (**Figure 2D**). IL-17F and IL-22, involved in mucosal immunity and tissue repair, were more abundant in non-diabetic mild patients, supporting efficient barrier defense and viral control. IL-12p70, which promotes cellular immunity, further suggests enhanced viral clearance in non-diabetics. IL-8, a neutrophil chemoattractant, was elevated in non-diabetic severe cases, implying neutrophil-driven inflammation may contribute to severity in these individuals. Conversely, diabetic severe patients exhibited impaired neutrophil activity, potentially allowing persistent viral replication. Notably, IL-10 was substantially increased in the Severe_DM and Mild_DM groups, possibly indicating immune exhaustion and serving as a prognostic marker in diabetic COVID-19 (**Figure 2D**).

To assign biological meaning to these proteomic alterations, we performed functional enrichment analyses. Gene Ontology (GO) enrichment analysis, integrating Biological Process (BP), Cellular Component (CC), and Molecular Function (MF), revealed distinct functional landscapes among the five groups (**Figure 3A**; **Supplementary Figure 2**). As disease severity escalated in non-diabetic patients (Mild to Severe), differential proteins were predominantly enriched in complement activation, immunoglobulin processes, B cell activation, etc. (**Figure 3A**). In diabetic patients, the Mild_DM group already exhibited marked enrichment in insulin related pathways, neutrophil degranulation, and this pro-inflammatory phenotype intensified in the Severe_DM group(**Figure 3A**). The cellular component analysis further mapped these proteins to extracellular exosome, platelet alpha granule, indicating systemic perturbations in circulating effector molecules.

**Figure 3:**
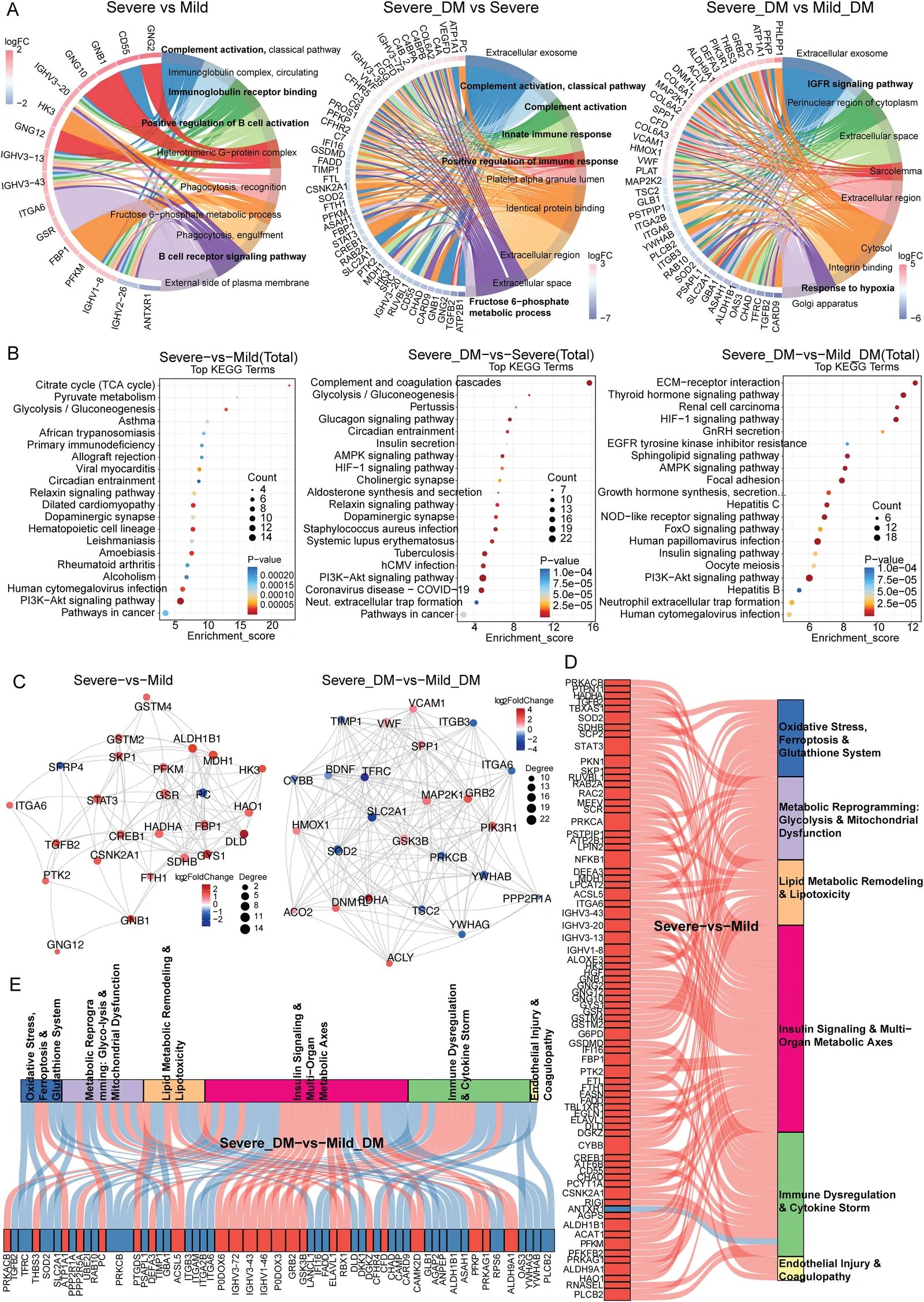
Functional annotation and network analysis of dysregulated proteins in SARS-CoV-2-infected patients with or without type 2 diabetes (T2DM). **(A)** Integrated Gene Ontology (GO) enrichment analysis combining biological process (BP), cellular component (CC), and molecular function (MF) categories. Bubble color and size reflect enrichment significance; highlighted terms include complement activation, neutrophil degranulation, and coagulation, which are associated with DM and severe disease progression. **(B)** Kyoto Encyclopedia of Genes and Genomes (KEGG) pathway enrichment. Key pathways including PI3K-Akt and HIF-1 signaling are highlighted; node color indicates differential expression direction in the Severe_DM group, facilitating identification of core metabolic and disease-related pathways. **(C)** Top three core modules identified by the MCODE algorithm from the protein-protein interaction (PPI) network. Node color corresponds to differential expression patterns across clinical groups; module size reflects interaction density, highlighting central regulatory hubs. **(D-E)** Sankey diagram of pathway-specific protein set analysis. Predefined protein sets related to insulin resistance and cardiorenal injury are displayed; chord width reflects correlation strength of protein expression trends, visualizing organ damage-associated molecular signatures. The red blocks next to the gene name indicate up-regulation of the corresponding protein, blue ones exhibited down-regulation. GO, Gene Ontology; KEGG, Kyoto Encyclopedia of Genes and Genomes; PPI, protein-protein interaction; MCODE, Molecular Complex Detection; DM, diabetes mellitus; BP, biological process; CC, cellular component; MF, molecular function.

Complementing these findings, Kyoto Encyclopedia of Genes and Genomes (KEGG) pathway enrichment highlighted signaling networks progressively activated along disease severity and diabetic comorbidity (**Figure 3B**; **Supplementary Tables 6**-**S8**). The PI3K-Akt signaling pathway, HIF-1 signaling pathway, thyroid hormone signaling pathway and insulin signaling pathway were among the top enriched pathways in the Severe_DM group. This visualization intuitively demonstrated that certain hub proteins were shared across multiple immune and metabolic pathways, reflecting coordinated dysregulation.

To visualize the physical interactions underlying these pathways, we constructed protein-protein interaction (PPI) network from the STRING database (**Figure 3C**). The anti-viral response modes of non-DM and DM are different. Non-DM Severe group comparing with Mild group, the hub nodes include GSR, MDH1, STAT3, SDHB, HADHA, and others, all up-regulated, indicating coordinated activation of antioxidant defenses, mitochondrial metabolic reprogramming, and pro-inflammatory signaling during severe viral infection. Whereas, the core regulatory network of Severe_DM vs Mild_DM, defined by upregulated GSK3B, GRB2, MAP2K1, etc. and downregulated SLC2A1, YWHAB, etc. reflects dysregulated glycogen metabolism, hyperactive MAPK/ERK signaling, and impaired glucose uptake in diabetic severe infection (**Figure 3C**). This regulatory mode diverges fundamentally from non-diabetic severe infection, which centers on canonical complement activation and acute inflammatory responses rather than pre-existing metabolic dysregulation and maladaptive signaling rewiring, consistent with baseline insulin resistance and metabolic inflexibility in T2DM.

We then focused on the 6 types of pathways including Oxidative Stress, Ferroptosis & Glutathione System, Metabolic Reprogramming: Glycolysis & Mitochondrial Dysfunction, Lipid Metabolic Remodeling & Lipotoxicity, Insulin Signaling & Multi−Organ Metabolic Axes, Immune Dysregulation & Cytokine Storm, and Endothelial Injury & Coagulopathy to figure key modulators in DM patients (**Figure 3D**; **Supplementary Figure 2**). We found that bidirectional dysregulation of core effectors across converging pathways in Severe_DM vs Mild_DM—downregulated PRKCB, PLCB2, RPS6, TFRC, SOD2 (impairing antioxidant defense and iron homeostasis) and upregulated GRB2, RBX1, CAMK2D, PFKP, ALDH9A1, ACSL5 (driving glycolytic flux and fatty acid oxidation), delineates a maladaptive multi-pathway rewiring characteristic of diabetic severe infection (**Figure 3E**). In non-diabetic patients following SARS-CoV-2 infection, nearly all proteins mapped to the six core pathways of interest were upregulated, with only ANTXR1 downregulated—an alteration presumably involved in maintaining immune homeostasis. Collectively, these data demonstrate drastically divergent host response patterns between diabetic and non-diabetic individuals following viral infection.

### Integrated metabolomic profiling reveals progressive metabolic deregulation

Given that proteins ultimately regulate metabolic fluxes, we next integrated metabolomic profiling to capture the functional metabolic consequences of these proteomic shifts. Integrated metabolomic profiling combining LC-MS and GC-MS platforms comprehensively characterized metabolic perturbations across the five patient groups (**Figure 4**; **Supplementary Figure 3**). Quantitatively, bar charts of LC/GC-MS-derived differential metabolites (FDR < 0.05, |log_2_FC| ≥ 1) revealed progressive metabolic dysregulation. The DM-vs-Non_DM comparison identified 349 altered metabolites, confirming baseline metabolic perturbations in diabetes. Critically, Severe_DM-vs-Severe yielded the highest burden (409 metabolites), far exceeding the 275 changes in non-diabetic Severe-vs-Mild, indicating diabetes amplifies metabolic disruption specifically upon progression to critical illness rather than via additive chronic hyperglycemia effects. In contrast, Mild_DM-vs-Mild showed only 239 alterations, skewed toward upregulation (162 vs 77), distinct from the balanced dysregulation in advanced disease. Visualizing these global shifts, joint PLS-DA analysis integrating both LC-MS and GC-MS datasets yielded clear group separation in both 3D-score plot and 2D-score plot representations (**Figure 4B**). The separation along the principal components underscores a profound, diabetes-dependent rewiring of the metabolic landscape.

**Figure 4:**
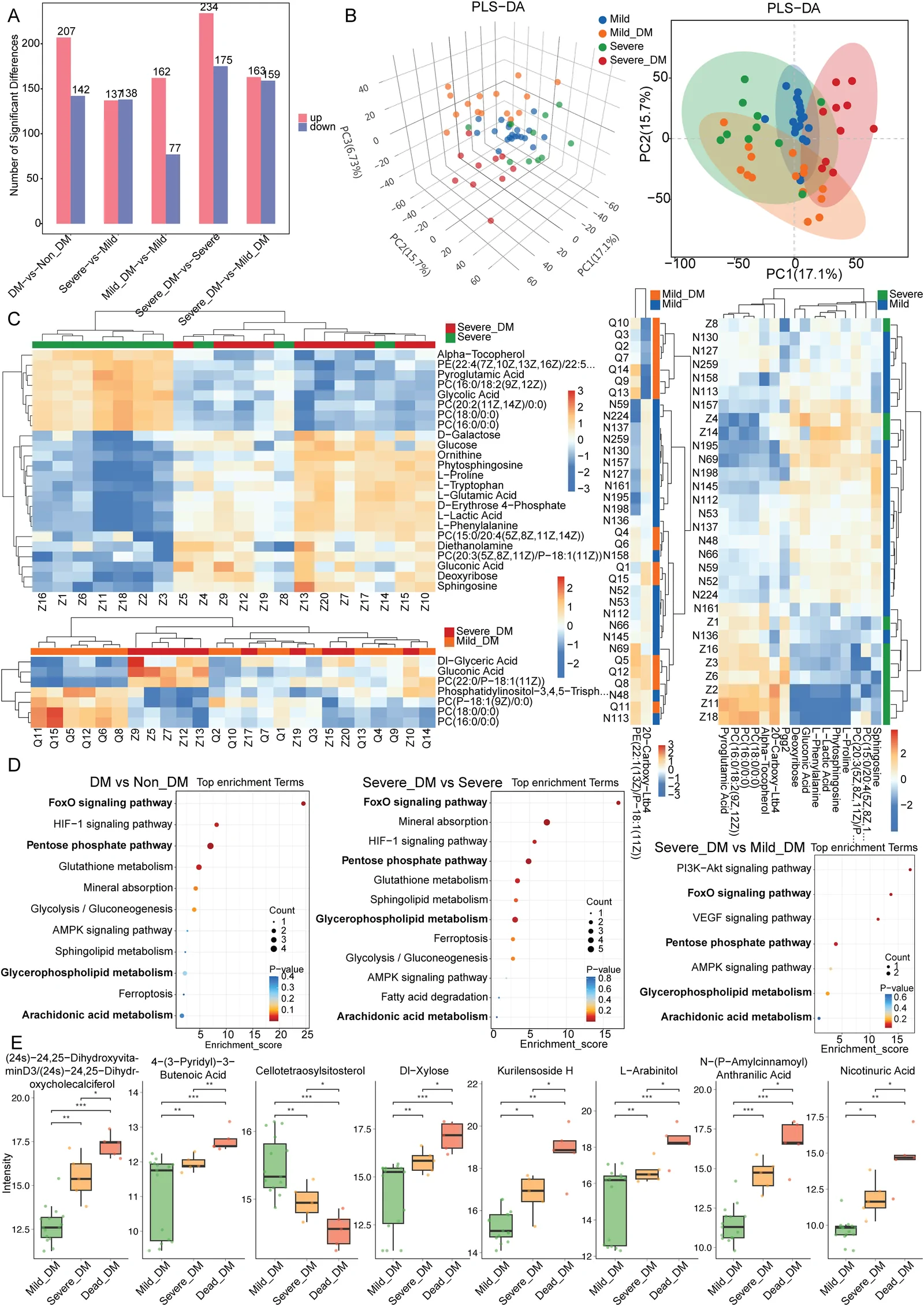
Integrated LC/GC-MS metabolomic profiling reveals metabolic dysregulation across disease severity and diabetic status. **(A)** Bar distribution of fold changes for differentially abundant metabolites across key clinical comparisons, providing an overview of global metabolic perturbation magnitude. **(B)** Multidimensional score plots (PLS-DA or PCA) derived from integrated dual-platform (LC-MS, GC-MS) data, illustrating the separation of metabolic profiles across clinical groups. **(C)** Hierarchical clustering heatmap of curated key metabolites (amino acids, fatty acids, glycolysis intermediates, etc.); row color intensity reflects relative metabolite abundance, and dendrograms depict metabolite co-regulation relationships and sample metabolic similarity. **(D)** KEGG pathway impact analysis focusing on core metabolic and disease-associated pathways; node visual attributes correspond to pathway enrichment significance and impact value, highlighting dysregulation linked to DM comorbidity and severe progression. **(E)** Box plots displaying metabolic trait gradients: left panel shows expression trends of representative metabolites across Mild_DM, Severe_DM, and Dead_DM groups; right panel details quantitative distributions of metabolites with extreme differential abundance in the Dead_DM group (e.g., lactic acid, specific sphingolipids) relative to the Severe_DM group. LC-MS, liquid chromatography-mass spectrometry; GC-MS, gas chromatography-mass spectrometry; PLS-DA, partial least squares discriminant analysis; PCA, principal component analysis; KEGG, Kyoto Encyclopedia of Genes and Genomes; DM, diabetes mellitus. Statistical significance was annotated as follows: *p < 0.05, **p < 0.01, ***p < 0.001.

Delving deeper into specific metabolite classes, unsupervised hierarchical clustering of key metabolites revealed distinct stratification patterns across four comparative panels (**Figure 4C**; **Supplementary Figure 3**). The segregation of non-diabetic Severe samples from Mild controls, defined by upregulation of the pro-inflammatory eicosanoid 20-Carboxy-LTB4, oxidative stress marker pyroglutamic acid, and antioxidant α-tocopherol, coupled with downregulation of the glycerophospholipid PC(20:3(5Z,8Z,11Z)/P-18:1(11Z)). In contrast, the adjacent panel contrasts Mild_DM and non-diabetic Mild samples, revealing a minimal yet stable signature of two consistently upregulated sphingolipids—phytosphingosine and sphingosine—with no significantly downregulated metabolites, reflecting early subclinical metabolic remodeling in diabetic hosts. Progressing to severe disease, the heatmap separates Severe_DM from Mild_DM cohorts, featuring robust upregulation of Dl-Glyceric Acid and Gluconic Acid alongside uniform downregulation of saturated phosphatidylcholines (PC(18:0/0:0), PC(16:0/0:0)), indicative of exacerbated glycolytic stress and membrane compositional shifts during diabetic disease progression. Finally, the panel distinguishes Severe_DM from non-diabetic Severe patients, with marked elevation of glycolytic end-products (L-lactic Acid, Gluconic Acid) and the aromatic amino acid L-phenylalanine in the diabetic severe cohort, demonstrating that diabetes amplifies metabolic dysregulation beyond the effects of severe infection alone. Collectively, these heatmaps trace a stepwise metabolic drift from baseline diabetic perturbations to fulminant dysregulation in severe diabetic viral pneumonia.

To translate these metabolite alterations into pathway-level insights, KEGG pathway analysis identified key metabolic routes distinguishing the clinical groups (**Figure 4D**; **Supplementary Figure 3**; **Supplementary Tables 9**-**S11**). Across all comparisons, Arachidonic acid metabolism, Glycerophospholipid metabolism, and the AMPK signaling pathway were consistently enriched. Specifically, the DM vs Non-DM comparison was characterized by enrichment in FoxO signaling, HIF-1 signaling, and the Pentose phosphate pathway (**Figure 4D**; **Supplementary Table 9**). Upon progression to severe disease, the Severe_DM vs Severe comparison highlighted Ferroptosis, Sphingolipid metabolism, and Glycolysis/Gluconeogenesis emerged as dominant pathways, pointing to oxidative stress and membrane lipid remodeling as critical drivers of DM-related severity (**Figure 4D**; **Supplementary Table 10**). Lastly, the Severe_DM vs Mild_DM comparison highlighted PI3K-Akt signaling, VEGF signaling, and Glutathione metabolism, reflecting a coordinated stress response involving cell survival, angiogenesis, and redox homeostasis (**Figure 4D**; **Supplementary Table 11**).

To identify metabolites specifically predictive of lethality, we conducted focused box-plot analyses comparing Mild_DM, Severe_DM, and Dead_DM groups (**Figure 4E**; **Supplementary Figure 3**). A cluster of 8 metabolites—including (24s)-24,25-Dihydroxyvitamin D3, 4-(3-Pyridyl)-3-Butenoic Acid, Dl-Xylose, L-Arabinitol, Anthranilic Acid, and Nicotinuric Acid—displayed a consistent and statistically significant stepwise escalation and decline in Cellotetraosylsitosterol, from mild to severe to fatal outcomes. This progressive accumulation establishes a clear metabolic gradient paralleling clinical deterioration and suggests that disruptions in vitamin D metabolism, pyridine metabolism, and sterol/sphingolipid pathways may serve as metabolic signatures of mortality risk in diabetic patients with severe viral pneumonia.

### Integrated proteomic and metabolomic analysis reveals cooperative dysregulation in diabetic patients with severe viral pneumonia

Leveraging the proteomic and metabolomic datasets described above, we identified a comprehensive set of proteins and metabolites closely linked to critical COVID-19 in diabetic patients during severe viral infection (**Figure 5**; **Supplementary Figure 4**). A cornerstone of this integration, Spearman correlation analysis revealed graded shifts in co-regulation patterns across clinical strata, with each comparison presented as an independent sub-heatmap (**Figure 5A**). At the mild diabetic stage, the Mild_DM vs Mild sub-heatmap delineated a module linking redox regulators (GSTP1/P30837, PRDX1/Q99490), lysosomal modulators (LAMP1/P42765, SMPD3/Q9BYJ1), and coagulation factors (FGA/P02675, FGB/P02679) with 20-Carboxy-LTB4 and PE(22:1(13Z)/P-18:1(11Z)), marking early metabolic-immune dysregulation. In the absence of diabetes, the Severe vs Mild sub-heatmap exhibited a tight cluster of complement effectors (C9/P20020, C3/P50151, CFB/P09622) with 20-Carboxy-LTB4, pyroglutamic acid, and α-tocopherol, reflecting systemic inflammation and oxidative stress. However, in the presence of diabetes, the Severe_DM vs Severe sub-heatmap showed an intensified pathology, coupling complement/neutrophil proteins (C9/P20020, CFB/P09622, ELANE/P08237, PCSK9/Q9Y265) with L-lactic acid, sphingolipids (sphingosine, phytosphingosine), and oxidized species (pyroglutamic acid, glycolic acid) to define a diabetes-specific inflammatory-amplification loop. Ultimately, the Severe_DM vs Mild_DM sub-heatmap revealed the most extensive rewiring, where hypoxia-responsive EGLN1/Q9Y6K5 and glycolytic ALDOA/P36507 co-clustered with recurrent phosphatidylinositol-3,4,5-trisphosphate (PIP3) and saturated phosphatidylcholines (PC(22:0/P-18:1), PC(16:0/0:0), PC(18:0/0:0)) alongside gluconic acid and DL-glyceric acid. This gradient of dysregulation, from early metabolic priming to hypoxia-driven PIP3 accumulation and membrane saturation, underscores the progressive collapse of homeostatic networks in diabetic patients with severe viral pneumonia.

**Figure 5:**
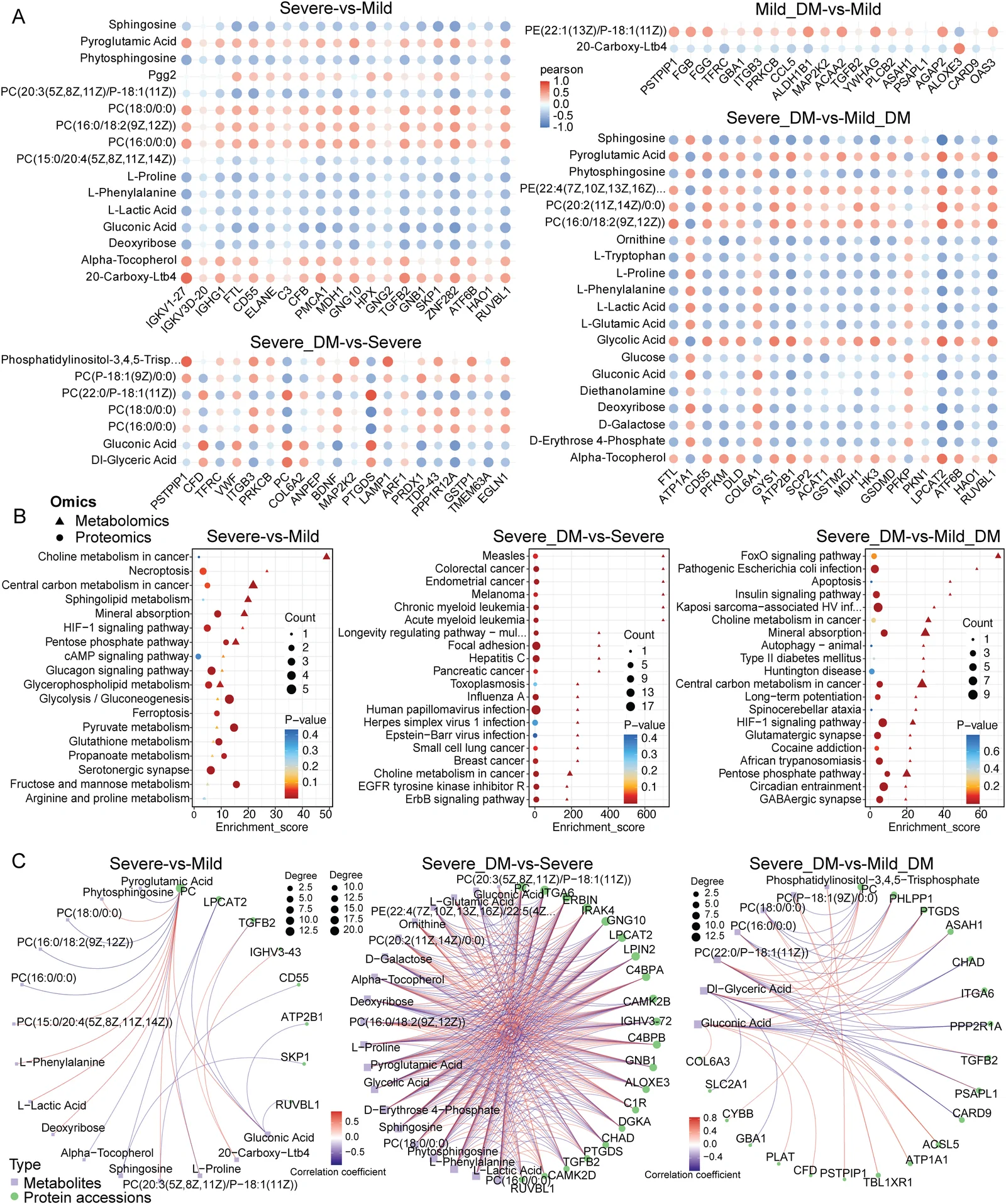
Integrated multi-omics analysis reveals cooperative dysregulation of proteins and metabolites in SARS-CoV-2-infected patients with type 2 diabetes. **(A)** Spearman correlation heatmap of differentially expressed proteins and metabolites. Color intensity encodes correlation coefficients; hierarchical clustering groups molecules into co-regulated modules, illustrating coordinated expression patterns across clinical groups. **(B)** Joint-pathway analysis via KEGG Mapper. Differentially abundant proteins and metabolites are mapped to reference KEGG pathways (e.g., HIF-1 signaling pathway); red and blue arrows denote significant upregulation and downregulation in the Severe_DM group relative to controls, respectively, visualizing convergent multi-omic regulation of core pathways. **(C)** Spearman correlation network of top-ranked differential molecules. Nodes represent proteins (green) and metabolites (light purple), with node size proportional to differential expression abundance; edge thickness corresponds to the strength of Spearman correlation, highlighting core regulatory hubs and interaction modules. Nodes represent proteins (red) and metabolites (green), with node size proportional to differential expression significance; edge thickness corresponds to the strength of Spearman correlation, highlighting core regulatory hubs and interaction modules. KEGG, Kyoto Encyclopedia of Genes and Genomes; DM, diabetes mellitus.

To validate the convergent nature of these findings, joint KEGG pathway mapping clarified mechanistic overlaps (**Figure 5B**; **Supplementary Figure 4**; **Supplementary Tables 12**-**S14**). For instance, in Severe vs Mild, upregulated PI3K-Akt and HIF-1 signaling coincided with downregulated fatty acid degradation, confirming a metabolic shift from oxidative phosphorylation to glycolysis (**Figure 5B**; **Supplementary Table 12**). Mirroring the single-omics data, Severe_DM vs Severe comparisons highlighted synergistic activation of glycolysis/gluconeogenesis and the AGE-RAGE axis, directly linking proteomic upregulation of glycolytic enzymes to lactate accumulation (**Figure 5B**; **Supplementary Table 13**). Conversely, Severe_DM vs Mild_DM dynamics centered on FoxO signaling and glutathione metabolism, reflecting progressive loss of redox homeostasis over the course of diabetic comorbidity (**Figure 5B**; **Supplementary Table 14**).

Complementing pathway-level insights, protein-metabolite interaction networks (retaining edges with r > 0.7, P < 0.01) illustrated topological differences across groups (**Figure 5C**; **Supplementary Figure 4**). As expected, the Severe vs Mild network was anchored by complement and coagulation hubs, consistent with thromboinflammation in non-diabetic severe disease. However, the Severe_DM vs Severe network shifted toward sphingolipid metabolism and energy-sensing molecules, marking a transition from pure inflammatory to mixed metabolic-inflammatory pathology. Most strikingly, the densest network was observed in Severe_DM vs Mild_DM comparisons, where cytoskeletal reorganization proteins and nucleotide metabolites emerged as core nodes—evidence that long-term diabetes remodels baseline homeostatic networks, rendering them vulnerable to irreversible collapse upon severe infection. Collectively, these integrated analyses define a core dysregulated protein-metabolite network driving poor outcomes in diabetic patients with severe viral pneumonia.

### Diabetes-specific molecular drivers of severe viral pneumonia

To isolate molecular alterations driven specifically by diabetes during severe disease progression, we defined the union_E signature as molecules uniquely dysregulated in Severe_DM-vs-Severe after excluding shared features present in Mild_DM-vs-Mild. Quantitatively, Venn analysis revealed 801 unique proteins (414) and metabolites (387) exclusive to Severe_DM-vs-Severe, outnumbering the differences in Mild_DM-vs-Mild (**Figure 6A**; **Supplementary Table 15**). This disparity indicates that diabetes-associated molecular perturbations are unmasked and amplified specifically upon progression to severe disease rather than representing additive effects of chronic hyperglycemia. At the correlative level, Spearman correlation heatmap of these union_E molecules identified a core co-regulated module linking redox regulators (e.g., SOD2/P04179, GOT2/P00505) to acylcarnitines and galactose derivatives, defining a characteristic metabolic dysregulation signature of diabetic severe infection (**Figure 6B**). Structurally, a correlation network delineated three core regulatory axes: the ion transporter SLC12A2/Q16512 exhibited the strongest positive correlation with the bioactive lipid DG(2:0/20:4(6Z,8E,10E,14Z)-2OH(5S,12R)/0:0) (r = 0.775, padj = 1.64 × 10^−^³), implicating impaired osmotic regulation; autophagy proteins MAP1LC3C/Q9BRF8 and ATG4C/Q13444 correlated negatively with gluconic acid and positively with 4-pyridoxic acid (r = −0.719, padj = 1.81 × 10^−^^5^; r = 0.594, padj = 2.88 × 10^−^²), reflecting disrupted autophagic flux; and the coagulation effector fibrinogen beta chain FGB/P19397 positively associated with the same DG species and negatively correlated with N-lactoyl-tryptophan (r = 0.683, padj = 1.37 × 10^−^²; r = −0.601, padj = 6.52 × 10^−^²), linking coagulation dysfunction to tryptophan metabolic reprogramming (**Figure 6C**). Directionally, Sankey diagram mapping clarified regulatory flows: 27 downregulated proteins including TSC2/P49815, FADD/Q13158, STAT3/P40763, and SOD2/P04179 converged on eight suppressed homeostatic pathways (e.g., autophagy, mTOR signaling), whereas upregulated signals (LALBA, CAMK2D, SIRT3) were restricted to carbon metabolism, galactose metabolism, HIF-1 signaling pathway and insulin signaling pathways (**Figure 6D**; **Supplementary Tables 16**, **S17**). This sharp asymmetry indicates that the primary pathological signature of diabetes during severe SARS-CoV-2 infection is broad suppression of most endogenous homeostatic networks, rather than activation of novel deleterious signaling programs, the observed upregulation of SIRT3 and related metabolic pathways likely represents a failed compensatory attempt to counteract systemic metabolic collapse.

**Figure 6:**
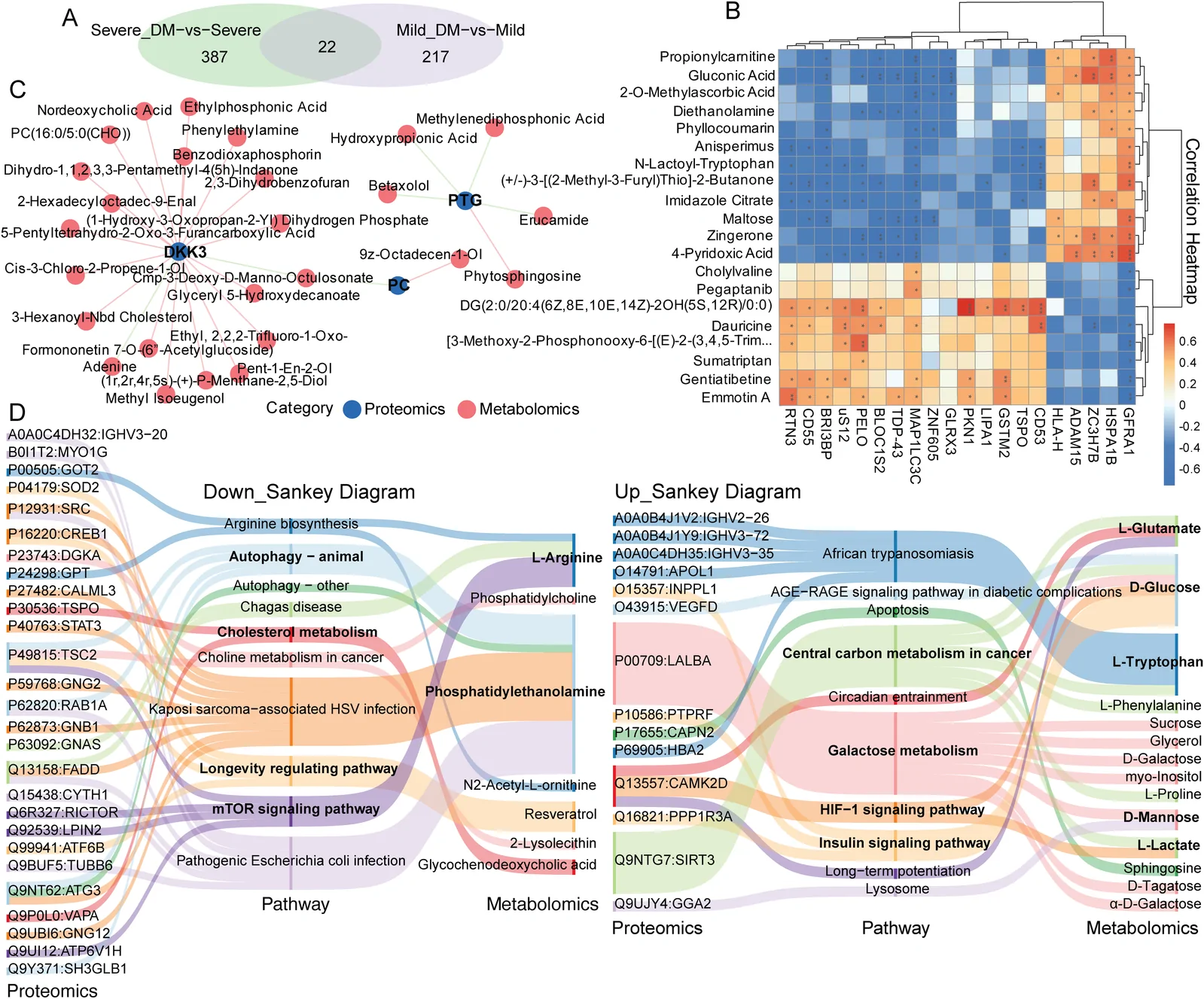
Union_E-defined diabetes-specific molecular signatures driving severe SARS-CoV-2 infection (see Supplementary **Figure 5** for extended analytical details). **(A)** Venn diagram delineating molecules uniquely dysregulated in the Severe_DM vs. Severe comparison after excluding features shared with the Mild_DM vs. Mild comparison, quantifying the set of molecular alterations specifically linked to diabetes-exacerbated severe disease. **(B)** Spearman correlation heatmap of union_E molecules, with color intensity encoding correlation coefficients; hierarchical clustering groups molecules into co-regulated modules, visualizing coordinated expression patterns across clinical groups. **(C)** Correlation network constructed from the most statistically significant union_E molecules; node size is proportional to differential expression significance, and edge thickness corresponds to Spearman correlation strength, highlighting core regulatory hubs underlying diabetic severe infection. **(D)** Bidirectional Sankey diagram mapping regulatory cascades across KEGG pathways; upper and lower streams denote upregulated and downregulated components, respectively, illustrating how upstream proteins/enzymes modulate downstream metabolites via defined signaling axes to influence disease progression. union_E, unique molecular signature of Severe_DM vs. Severe after excluding features shared with Mild_DM vs. Mild; KEGG, Kyoto Encyclopedia of Genes and Genomes; DM, diabetes mellitus.

### Multi-omics analysis identifies lethal risk factors

Finally, to delineate the molecular determinants of fatal outcomes, we compared the proteomic and metabolomic profiles of Dead_DM patients against those with Severe_DM (**Figure 7**). Hierarchical clustering heatmap of the top 50 differentially expressed proteins distinctly segregated the Dead_DM cohort from Severe_DM controls (**Figure 7A**). Notably, the Dead_DM signature was defined by concurrent upregulation of C2CD2L, NAA10, PI4KA, and CRIP1—reflecting dysregulated lipid trafficking, protein N-terminal acetylation, phosphoinositide signaling, and cytoskeletal remodeling—and downregulation of NRGN, ATRN, A4GALT, DAPPA, TBXAS1, and SMOC1, which collectively impair neurogenesis-associated signaling, thrombotic/thromboxane biosynthesis, extracellular matrix integrity, and immune homeostasis (**Figure 7A**) (17, 18).

**Figure 7:**
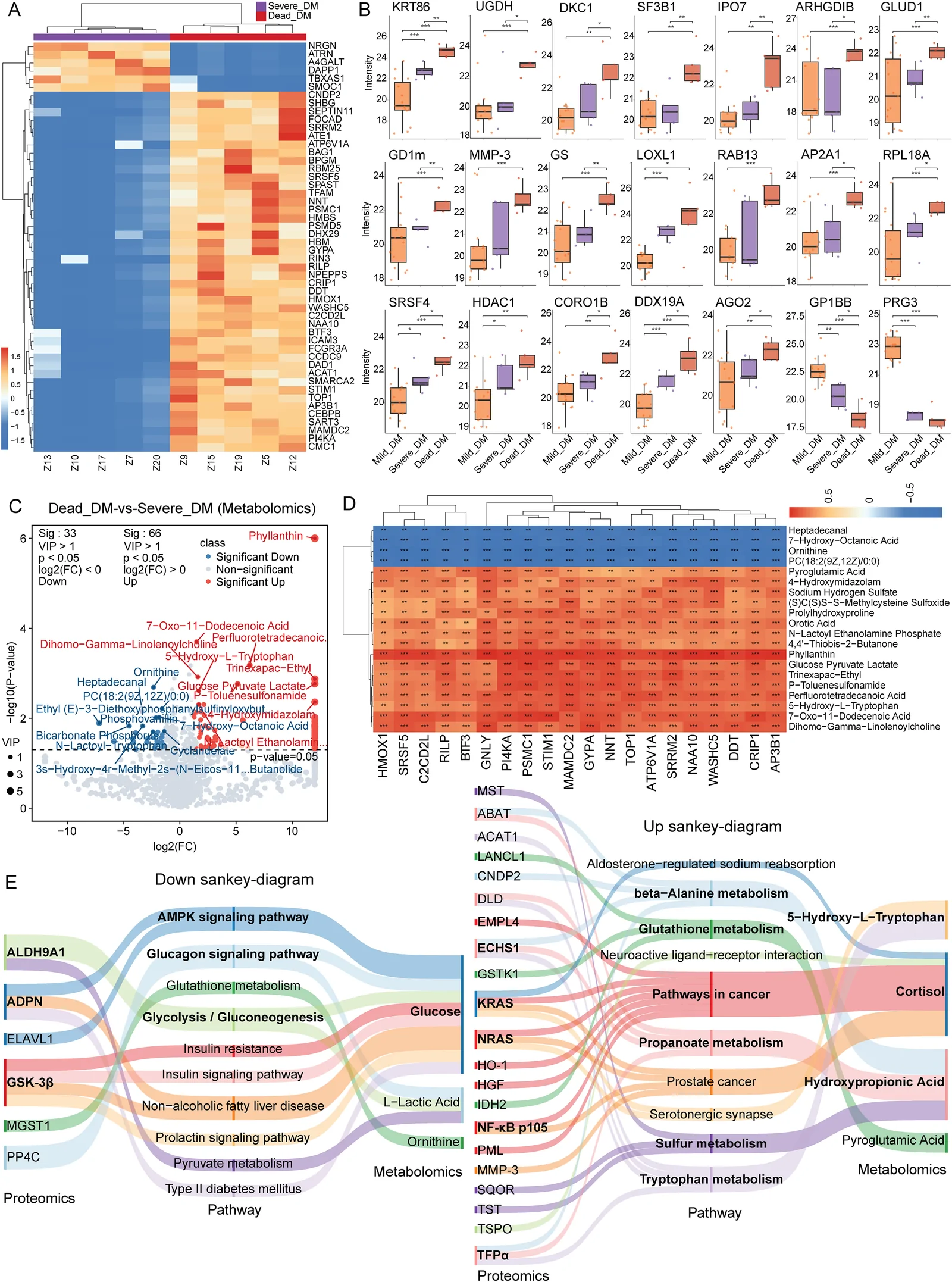
Integrated multi-omics profiling identifies lethal risk factors in patients with type 2 diabetes mellitus (T2DM) and severe SARS-CoV-2 infection. **(A)** Hierarchical clustering heatmap of top differentially expressed proteins between Dead_DM and Severe_DM groups; row color intensity reflects relative protein abundance, and dendrograms depict co-expression relationships to highlight fatal-disease-specific signatures. **(B)** Box plots validating expression trends of key dysregulated proteins in Dead_DM versus Severe_DM cohorts; box bounds denote interquartile ranges, with whiskers extending to minima/maxima within 1.5× interquartile range. Statistical significance was annotated as follows: *p < 0.05, **p < 0.01, ***p < 0.001. **(C)** Volcano diagram of differentially abundant metabolites distinguishing Dead_DM from Severe_DM patients. Rows represent individual metabolites, columns represent patient samples, and color intensity reflects relative metabolite abundance. **(D)** Spearman correlation analysis of DEPs and differential metabolites (|r| > 0.6, P < 0.01; * denotes statistical reliability, with more asterisks indicating higher correlation confidence). The red module captures positive correlations between these DEPs and metabolites. The blue module captures negative correlations between the same DEPs and depleted metabolites. **(E)** Bidirectional Sankey diagrams mapping directional regulatory cascades across KEGG pathways (P < 0.05). The downregulated Sankey delineates convergent suppression of 10 core homeostatic pathways and related DEPs and metabolites. The upregulated Sankey reveals coordinately activate 10 maladaptive stress pathways. T2DM, type 2 diabetes mellitus; KEGG, Kyoto Encyclopedia of Genes and Genomes; Dead_DM, fatal diabetic COVID-19 cohort; Severe_DM, severe diabetic COVID-19 cohort. Keratin, type II cuticular Hb6, KRT86; UDP-glucose 6-dehydrogenase, UGDH; H/ACA ribonucleoprotein complex subunit DKC1, DKC1; Splicing factor 3B subunit 1, SF3B1; Importin-7, IPO7; Rho GDP-dissociation inhibitor 2, ARHGDIB; Glutamate dehydrogenase 1, mitochondrial, GLUD1; Stromelysin-1, MMP-3; Glutamine synthetase, GS; Protein-lysine 6-oxidase, LOXL1; Ras-related protein Rab-13, RAB13; AP-2 complex subunit alpha-1, AP2A1; 60S ribosomal protein L18a, RPL18A; Serine/arginine-rich splicing factor 4, SRSF4; Histone deacetylase 1, HDAC1; Coronin-1B, CORO1B; ATP-dependent RNA helicase DDX19A, DDX19A; Protein argonaute-2, AGO2; Platelet glycoprotein Ib beta chain, GP1BB; Proteoglycan 3, PRG3.

To identify candidate biomarkers associated with disease progression in diabetic patients with severe COVID-19, we performed quantitative proteomic profiling. Box plots (**Figure 7B**) delineate a clear stepwise expression trajectory of DEPs across the clinical continuum from Mild_DM to Severe_DM to Dead_DM. The majority of these DEPs, including KRT86, UGDH, DKC1, SF3B1, IPO7, ARHGDIB, GLUD1, MMP-3, GS, LOXL1, RAB13, AP2A1, RPL18A, SRSF4, HDAC1, CORO1B, DDX19A, and AGO2, exhibited a progressive, stepwise increase in abundance concomitant with worsening disease severity. In contrast, platelet glycoprotein Ib beta chain (GP1BB) and proteoglycan 3 (PRG3) displayed a consistent, stepwise decline across the same clinical gradient, with the lowest expression levels recorded in the Dead_DM cohort. The progressive elevation of MMP-3, LOXL1, and GLUD1, coupled with the stepwise decline of GP1BB and PRG3, warrants close monitoring in diabetic patients following infection. As MMP-3 play central role in extracellular matrix degradation and inflammatory amplification, LOXL1 and GLUD1 further implicates vascular remodeling and metabolic reprogramming as synergistic mechanisms exacerbating disease severity in this high-risk population (19, 20). Conversely, the progressive depletion of GP1BB, an essential subunit for PLT adhesion, paralleled clinical deterioration and likely contributes to the thrombotic/hemorrhagic diathesis characteristic of fatal diabetic COVID-19 (21).

Parallel metabolomic profiling revealed distinct alterations in Dead_DM patients (**Figure 7C**). The fatal metabolic signature exhibited a striking substrate misalignment: significant depletion of free utilizable energy substrates and homeostatic metabolites, paradoxically accompanied by accumulation of a glycolytic flux-related adduct and a panel of toxic pro-inflammatory effectors. Depletion of free energy and homeostatic reserves. Dead_DM patients showed significant declines in glucose, L-lactic acid, the urea cycle intermediate ornithine, alongside depletion of the membrane-stabilizing lysophosphatidylcholine PC(18:2) and the branched-chain amino acid L-leucine. This coordinated loss implicates concurrent failures in peripheral substrate utilization, urea cycle flux, and mitochondrial membrane integrity, reflecting systemic erosion of metabolic resilience (22, 23).

Notably, paradoxical accumulation of a glycolytic adduct and toxic mediators. While free glucose and lactate were depleted, the combined feature Glucose Pyruvate Lactate, likely representing a specific mass-spectrometric adduct or total glycolytic flux pool, was significantly elevated in Dead_DM patients. This reciprocal pattern may unveil a terminal deadlock: the adduct pool passively accumulates as a byproduct of ongoing systemic glycolysis, whereas the concurrent decline in free glucose and lactate signifies the exhausted capacity of peripheral tissues to extract and oxidize these fuels, driven by refractory insulin resistance and mitochondrial failure, a scenario mirrored by the pronounced clinical glucose lability in fatal cases (**Figure 1B**). Simultaneously, the fatal metabolome was marked by robust accumulation of oxidative stress markers (e.g., pyroglutamic acid), lipid peroxidation products (e.g., 7-oxo-11-dodecenoic acid), and pro-inflammatory lysophospholipids (e.g., dihomo-gamma-linolenoylcholine), which together create a toxic microenvironment accelerating multiorgan collapse (24, 25).

To bridge the correlation pattern between DEPs and differential metabolites in Dead_DM versus Severe_DM patients, correlation analysis was performed (**Figure 7D**), identifying two co-regulated modules via hierarchical clustering (|r| > 0.6, P < 0.01). The red module captured significant positive correlations linking proteins (C2CD2L, NAA10, PI4KA, CRIP1, COXM1, MAMDC2, etc.) to metabolites (e.g., dihomo-γ-linolenoylcholine, phyllanthin). Conversely, the blue module revealed significant negative correlations between these proteins and metabolites (e.g., ornithine, PC(18:2), heptadecanal). Notably, the most reliable correlations were observed among the upregulated proteins. This reciprocal correlation pattern, where elevated lethal-associated proteins track with toxic metabolite accumulation while concurrently depleting protective metabolic pools, defines a characteristic molecular signature of fatal diabetic COVID-19, linking pathogenic protein overexpression to the exhaustion of homeostatic reserves.

To map the directional flow of these molecular events, we constructed Sankey diagrams tracing regulatory cascades across KEGG pathways in Dead_DM versus Severe_DM patients (P < 0.05, **Figure 7E**). The downregulated Sankey delineated convergent suppression of 10 homeostatic pathways, including AMPK signaling, glucagon signaling, and Glycolysis/Gluconeogenesis, by 6 downregulated proteins (ALDH9A1, ADPN, GSK-3β, MGST1) and 3 depleted metabolites (glucose, L-lactic acid, ornithine), reflecting coordinated failure of energy sensing, glycemic control, and central carbon metabolism. Conversely, the upregulated Sankey revealed that 21 elevated proteins (ECHS1, KRAS, NRAS, NF-κB p105, TFPα) and 4 accumulated metabolites (cortisol, 5-hydroxy-L-tryptophan, hydroxypropionic acid, pyroglutamic acid) coordinately activated 10 distinct pathways spanning pathways in cancer, beta-alanine metabolism, glutathione metabolism, propanoate metabolism, and sulfur metabolism (**Figure 7E**). This reciprocal regulatory pattern, where loss of protective molecules disables endogenous metabolic homeostasis while gain of toxic/inflammatory effectors hyperactivates maladaptive stress pathways, directly extends the bidirectional co-regulation signature defined in **Figure 7D**, defining a lethal molecular logic in which failed protective programs and pathological overactivation synergize to drive irreversible multiorgan collapse in fatal diabetic COVID-19.

## Discussion

Diabetic patients exhibit a markedly elevated risk of severe outcomes following SARS-CoV-2 infection. SARS-CoV-2 targets metabolic organs such as the pancreas and liver via ACE2 receptors, disrupt systemic energy homeostasis, accelerate disease deterioration, and even induce new-onset diabetes, thereby forming a bidirectional vicious cycle linking viral infection and metabolic disturbance (26, 27). Although multiple clinical risk factors for poor prognosis in diabetic COVID-19 patients have been identified, the precise molecular mechanisms driving heterogeneous disease progression and prognostic disparities among diabetic individuals remain incompletely defined. Our integrated analysis of clinical phenotypes, 4D-DIA proteomics, and dual-platform metabolomics provides a granular molecular portrait of severe COVID-19 in patients with T2DM, derived from a unique cohort recruited during the December 2022–February 2023 Omicron epidemic peak in China. This temporal specificity renders the dataset a rare resource for understanding diabetes-specific vulnerabilities to viral infection, as matched prospective cohorts are no longer feasible amid rapidly evolving SARS-CoV-2 variants.

Consistent with prior clinical observations, diabetic patients in our cohort experienced markedly worse outcomes than their non-diabetic counterparts, characterized by progressive thrombocytopenia, elevated neutrophil counts, and a high burden of polymicrobial and fungal secondary infections (28, 29). Biologically, chronic hyperglycemia in T2DM establishes a state of systemic immune dysfunction, which creates a permissive niche for secondary pathogens, meaning that polymicrobial and fungal coinfections are not arbitrarily assigned external exposures but downstream manifestations of diabetes-related immune paralysis. At the same time, removing the variance attributable to secondary infection would be analogous to adjusting for ARDS or microthrombosis when studying severe COVID-19, it would artificially strip away a core component of the diabetic disease phenotype we aim to characterize. Therefore, we interpret these coinfections not as statistical confounders but as integral features of severe diabetic COVID-19, as they were tightly linked to fatal outcomes and reflect the impaired immune surveillance inherent to chronic hyperglycemia (30). Circulating cytokine profiling supported this interpretation as non-diabetic patients with mild disease mounted robust early antiviral responses, with elevated IL-17F, IL-22, and SARS-CoV-2-specific antibody titers, whereas diabetic groups exhibited blunted protective cytokine signaling alongside elevated IL-6 and IL-10, consistent with immune exhaustion. This immunologic disconnect likely delays viral clearance and creates a permissive niche for secondary pathogens, compounding end-organ injury (31). Importantly, by deliberately retaining polymicrobial and fungal co-infections as integral features rather than statistical confounders, our study underscores that diabetes-induced mucosal immune impairment actively shapes disease trajectory rather than merely correlating with it.

Our multi-omic data extend these clinical observations to reveal a core “metabolic vulnerability” signature in diabetic severe disease, defined by coordinated dysregulation across six interconnected pathways, including oxidative stress, ferroptosis and glutathione homeostasis, glycolytic reprogramming and mitochondrial dysfunction, lipid remodeling and lipotoxicity, insulin signaling and multi-organ metabolic crosstalk, and endothelial injury with coagulopathy (**Figures 3D, E**). A key insight from our union_E analysis—which isolated molecules uniquely altered in Severe_DM versus Severe comparisons after excluding baseline differences between Mild_DM and Mild groups—was that diabetes-associated molecular perturbations are not static, additive effects of chronic hyperglycemia, but are instead unmasked and amplified specifically upon progression to critical illness. The 801 unique union_E molecules identified here, supporting the hypothesis that metabolic inflexibility primes diabetic hosts for catastrophic failure upon viral challenge. Central to this dysregulation was the upregulation of glycosylation machinery proteins (including NAA10, PI4KA, and C2CD2L), which may act as a molecular hinge linking hyperglycemia-driven hexosamine flux to downstream pathologies including vascular remodeling, lipid peroxidation (exemplified by accumulation of 7-oxo-11-dodecenoic acid), and saturated phosphatidylcholine depletion (e.g., PC(18:0/0:0)). This cascade likely establishes a self-reinforcing cycle of metabolic dysfunction, immune exhaustion, and secondary infection that may contribute to disease progression (32, 33).

Notably, we observed a clear molecular gradient tracking with clinical deterioration from Mild_DM to Severe_DM to Dead_DM. Proteins including MMP-3, LOXL1, and the glycosylation regulators showed progressive elevation with worsening severity, while platelet adhesion mediator GP1BB and immune modulator PRG3 declined consistently across the same continuum. The stepwise loss of GP1BB is particularly noteworthy, as an essential subunit of the PLT GPIb-IX-V complex, its depletion may underlie the paradoxical thrombotic and hemorrhagic diathesis characteristic of fatal diabetic COVID-19 (21). Its progressive depletion may underlie the paradoxical coexistence of thrombotic and hemorrhagic diathesis in fatal cases, excessive platelet consumption in microthrombi coupled with impaired adhesion reserve, tilting hemostasis toward an unstable, consumptive coagulopathy (34). On the metabolomic level, nine analytes including Dl-Xylose, L-arabinitol, and anthranilic acid exhibited a similar stepwise escalation with fatal outcomes, suggesting potential utility as dynamic markers of disease progression (34, 35) Intriguingly, metabolomic analysis further revealed a substrate misalignment in fatal cases: while the combined adduct Glucose Pyruvate Lactate accumulated, glucose and lactate were paradoxically depleted, pointing to a terminal metabolic deadlock in which peripheral tissues lose capacity to utilize circulating fuels, mirroring the clinical glucose lability in Dead_DM patients (**Figure 1B**) (36). Integrated regulatory analysis further delineated a “two-hit” lethal logic: the concurrent suppression of core homeostatic regulators (e.g., STAT3, TSC2, FADD) was associated with impaired adaptive pathways including AMPK signaling and autophagy, whereas the selective upregulation of stress-response effectors (e.g., LALBA, CAMK2D, SIRT3), coupled with accumulation of toxic metabolites such as cortisol and pyroglutamic acid, correlated with activation of maladaptive stress programs including glutathione and propanoate metabolism (17, 37) This reciprocal failure of protective programs and pathological overactivation likely drives the irreversible multiorgan collapse observed in fatal cases, though causal relationships remain to be confirmed via functional studies. Notably, several stress-responsive mediators exhibited stage-dependent directional shifts. For instance, GSK3B and ALDH9A1 were upregulated in Severe_DM versus Mild_DM, yet downregulated in Dead_DM versus Severe_DM. We interpret this reversal as a transition from compensatory stress adaptation to homeostatic exhaustion, with initial upregulation likely represents an attempted countermeasure against hyperglycemia-induced injury and MAPK/ERK hyperactivation, whereas subsequent suppression in fatal cases indicates that such compensatory reserves are ultimately overwhelmed, consistent with a terminal collapse of metabolic resilience rather than mere pathway inhibition (38, 39).

In sum, these findings offer several hypothesis-generating directions for future clinical research, though we emphasize that all conclusions are derived from an observational study and require rigorous validation in independent cohorts and functional experiments. First, the graded dysregulation of candidates across the Mild_DM→Severe_DM→Dead_DM continuum suggests potential biomarker applications that may differ across clinical junctures. MMP-3 and LOXL1 exhibited progressive elevation from mild to severe disease, raising the possibility that they could serve as progression markers heralding transition to critical illness, pending prospective evaluation. In contrast, ornithine and PC(18:2) displayed sharp declines specifically in Dead_DM versus Severe_DM, hinting that they might more faithfully track homeostatic reserve exhaustion and proximity to fatal outcomes. The concurrent decline of PRG3 alongside GP1BB, reflecting broad erosion of both platelet adhesive function and eosinophil-derived immune modulation, may indicate multidimensional depletion of protective reserves in fatal cases, though the functional consequences of these associations remain to be established.

Second, rather than relying on single molecules, our correlation network analysis (**Figure 7D**) raises the possibility that combining orthogonal markers, such as the protein pair GP1BB/MMP-3 with the metabolite Dl-Xylose, might offer improved predictive performance by capturing distinct biological dimensions (hemostatic attrition, extracellular matrix remodeling, and metabolic dysregulation). In terms of orthogonality to established clinical tools, CURB-65 and NEWS2 are constructed from physiological parameters (e.g., confusion, urea, respiratory rate, blood pressure, age, oxygen saturation) that primarily reflect acute organ dysfunction and physiological decompensation (40, 41). The biomarkers proposed here, by contrast, capture molecular processes entirely outside the scope of these scores: GP1BB depletion signifies exhaustion of platelet adhesive reserve, MMP-3 elevation drives maladaptive extracellular matrix turnover and inflammatory amplification, and Dl-Xylose accumulation indicates glycolytic flux perturbation and systemic metabolic dysregulation. We therefore hypothesize that a multi-panel integrating GP1BB, MMP-3, and Dl-Xylose could complement CURB-65 or NEWS2 in risk-stratifying diabetic patients with COVID-19. Formal validation of this additive value, via metrics such as category-free NRI, integrated discrimination improvement, or combined ROC/AUC analyses, will require prospective, multi-center cohorts with serial clinical scoring and sufficient fatal/critical endpoints to define robust cutoff values across diverse populations.

Third, our data highlight the glycosylation machinery, lipid metabolic remodeling, and antioxidant defense systems as areas of interest for future therapeutic exploration. Specifically, the progressive elevation of MMP-3 and LOXL1 in severe diabetic patients invites speculation that pharmacological inhibition of matrix remodeling, using repurposed agents such as doxycycline (a broad-spectrum MMP inhibitor) or LOX-specific blockers, might merit investigation in preclinical models of diabetic viral pneumonia, though such hypotheses require systematic testing before any clinical translation can be considered. Additionally, our continuous monitoring data suggest that glucose variability, particularly hypoglycemic episodes, was more closely associated with fatal outcomes than mean glucose levels, hinting that glycemic stability may warrant greater attention in acute infection management, although causal inferences cannot be drawn from this observational dataset.

Finally, the high prevalence of secondary infections in our severe and fatal cohorts highlights the need for further clinical research into tailored antimicrobial strategies and the potential interplay between infection control and metabolic management in this vulnerable population.

## Limitations

A main limitation for this study is the modest sample size of the Dead_DM subgroup, which should be enlarged in multi-center in the future if more funding is available. Besides, this (December 2022–February 2023), when circulating strains were predominantly BA.5.2 and BF.7 sublineages, and most participants had received inactivated COVID-19 vaccines per national immunization policies and had not been infected before. While acute respiratory viral stressors induced by different Omicron subvariants may cause minor variability in outcomes among the general population, the core pathophysiological vulnerabilities of T2DM patients, including chronic hyperglycemia, low-grade inflammation, endothelial dysfunction, and dysregulated coagulation, remain invariant and clinically decisive. Thus, the molecular signatures identified herein (e.g., progressive GP1BB depletion, MMP-3 upregulation, and metabolic dysregulation) represent intrinsic, diabetes-specific responses to viral insult, rather than artifacts of transient variant circulation, may need to undergo functional validation *in vitro* or *in vivo*. The absence of an independent external validation cohort further tempers the immediate generalizability of our conclusions to populations with different ethnic backgrounds or mRNA vaccine exposure histories. In addition, several potential confounders were not measured, including exact HbA1c at admission (although glycated albumin was assessed, standardized HbA1c was not available for all patients), insulin use prior to admission, and SARS-CoV-2 viral load (which was not quantified per the Diagnosis and Treatment Protocol Version 10 criteria applied during the study period). Nevertheless, this cohort provides a unique, time-sensitive snapshot of a high-risk demographic frequently underrepresented in global multi-omics studies, and our findings offer a critical foundation for future risk-stratification frameworks targeting diabetic patients facing viral epidemics.

## Data Availability

The datasets presented in this study can be found in online repositories. The names of the repository/repositories and accession number(s) can be found below: http://www.proteomexchange.org/, PXD066966.

## Supplementary material

Supplementary Table 1 Overlap of differentially expressed proteins (DEPs) across all five clinical comparison groups, corresponding to the Venn diagram in **Figure 2C**. Columns include comparison pair, count of upregulated/downregulated DEPs, and shared DEP identifiers.

Supplementary Table 2 DEPs identified in the Severe vs. Mild comparison. Columns include protein name, UniProt accession, log_2_ fold change (log_2_FC), false discovery rate (FDR), and normalized abundance. Corresponding volcano plot is presented in **Figure 2B**.

Supplementary Table 3 DEPs identified in the Mild_DM vs. Mild comparison. Columns follow the same structure as **Supplementary Table 2**. Corresponding volcano plot is presented in **Figure 2B**.

Supplementary Table 4 DEPs identified in the Severe_DM vs. Severe comparison. Columns follow the same structure as **Supplementary Table 2**. Corresponding volcano plot is presented in **Figure 2B**.

Supplementary Table 5 DEPs identified in the Severe_DM vs. Mild_DM comparison. Columns follow the same structure as **Supplementary Table 2**. Corresponding volcano plot is presented in **Figure 2B**.

Supplementary Table 6 KEGG pathway enrichment results for DEPs in the Mild_DM vs. Mild comparison. Columns include pathway name, enrichment factor, p-value, FDR, and count of enriched proteins. Corresponding bubble plot is presented in **Figure 3B**.

Supplementary Table 7 KEGG pathway enrichment results for DEPs in the Severe_DM vs. Severe comparison. Columns follow the same structure as **Supplementary Table 6**. Corresponding bubble plot is presented in **Figure 3B**.

Supplementary Table 8 KEGG pathway enrichment results for DEPs in the Severe_DM vs. Mild_DM comparison. Columns follow the same structure as **Supplementary Table 6**. Corresponding bubble plot is presented in **Figure 3B**.

Supplementary Table 9 KEGG pathway enrichment results for differentially abundant metabolites in the DM vs. Non_DM comparison. Columns include pathway name, impact value, p-value, FDR, and count of enriched metabolites. Corresponding bubble plot is presented in **Figure 4D**.

Supplementary Table 10 KEGG pathway enrichment results for differentially abundant metabolites in the Severe_DM vs. Severe comparison. Columns follow the same structure as **Supplementary Table 9**. Corresponding bubble plot is presented in **Figure 4D**.

Supplementary Table 11 KEGG pathway enrichment results for differentially abundant metabolites in the Severe_DM vs. Mild_DM comparison. Columns follow the same structure as **Supplementary Table 9**. Corresponding bubble plot is presented in **Figure 4D**.

Supplementary Table 12 Integrated multi-omics KEGG pathway mapping results for the Severe vs. Mild comparison. Columns include pathway name, mapped differential proteins/metabolites, and regulation direction (up/down). Corresponding joint-pathway analysis is presented in **Figure 5B**.

Supplementary Table 13 Integrated multi-omics KEGG pathway mapping results for the Severe_DM vs. Severe comparison. Columns follow the same structure as **Supplementary Table 12**. Corresponding joint-pathway analysis is presented in **Figure 5B**.

Supplementary Table 14 Integrated multi-omics KEGG pathway mapping results for the Severe_DM vs. Mild_DM comparison. Columns follow the same structure as **Supplementary Table 12**. Corresponding joint-pathway analysis is presented in **Figure 5B**.

Supplementary Table 15 Overlap of differentially expressed molecules in the Severe_DM vs. Severe comparison after excluding features shared with the Mild_DM vs. Mild comparison (union_E signature), corresponding to the Venn diagram in **Figure 6A**. Columns include molecule type (protein/metabolite), identifier, and regulation direction.

Supplementary Table 16 Downregulated KEGG pathways enriched in the union_E signature, corresponding to the downregulated flow in the Sankey diagram (**Figure 6D**). Columns include pathway name, enrichment metrics, and associated downregulated proteins/metabolites.

Supplementary Table 17 Upregulated KEGG pathways enriched in the union_E signature, corresponding to the upregulated flow in the Sankey diagram (**Figure 6D**). Columns include pathway name, enrichment metrics, and associated upregulated proteins/metabolites.

Supplementary Table 18 Top 50 differentially expressed proteins distinguishing the Dead_DM and Severe_DM groups. Columns include protein name, UniProt accession, log_2_FC, FDR, and normalized abundance across all samples. Corresponding heatmap and box plots are presented in **Figure 7A** and 7B.

Supplementary Figure 1 Muti-omics confounder adjustment analyses and quality control analysis supporting the proteomic profiling. **(A, C)** Assessment of confounding effects on protein expression and metabolites. Spearman rank correlation matrix of clinical covariates (age, BMI, sex, comorbidities, vaccine type, and dose number). All correlation coefficients clustered within the −0.5 to 0.5 range, indicating no strong inter-variable correlations or multicollinearity. **(B, D)** Box plot showing the variation contribution of each covariate and post-correction residual variation in omics datasets. **(E)** Quality control metrics for 4D-DIA mass spectrometry, including intensity distributions and coefficient of variation across replicates, confirming the reliability of the quantitative proteomic dataset. **(F)** Volcano plots illustrating differentially expressed proteins (DEPs) across additional pairwise comparisons beyond those presented in **Figure 2B**. Red and blue dots denote significantly upregulated and downregulated proteins, respectively (FDR < 0.05, |log_2_FC| ≥ 1). **(G)** Venn diagrams depicting the overlap of DEPs among all five clinical groups, highlighting shared and group-specific molecular signatures. DEPs, differentially expressed proteins; FDR, false discovery rate; FC, fold change.

Supplementary Figure 2 Supplementary functional annotation analyses supporting **Figure 3**. **(A)** GO cellular component (CC) enrichment analysis. The plot displays the cellular localization distribution of differentially expressed proteins, with bubble color encoding enrichment significance and bubble size reflecting the number of enriched molecules; key compartments include extracellular exosome and platelet alpha granule. **(B)** Circular chord diagram illustrating the interrelationship between key differential proteins and their enriched GO/KEGG terms. The outer ring lists functional categories, inner chords connect individual proteins to their associated terms, and chord color denotes up- (red) or down- (blue) regulation trends, visualizing the coordinated functional architecture of dysregulated proteins. **(C-D)** Extended KEGG pathway enrichment dot plots across additional comparisons, with dot color indicating enrichment significance and dot size reflecting the count of enriched molecules, complementing the focused pathway presentation in **Figure 3B**. **(E)** Full protein-protein interaction (PPI) network topology prior to MCODE module extraction, with node color representing differential expression direction, providing the global interaction context for the top 3 core modules highlighted in **Figure 3C**. **(F)** Sankey diagram of pathway-specific protein set analysis. The red blocks next to the gene name indicate up-regulation of the corresponding protein, blue ones exhibited down-regulation.GO, Gene Ontology; KEGG, Kyoto Encyclopedia of Genes and Genomes; PPI, protein-protein interaction; CC, cellular component; MCODE, Molecular Complex Detection.

Supplementary Figure 3 Supplementary metabolomic profiling analyses supporting **Figure 4**. **(A)** Quality control metrics for integrated LC-MS and GC-MS platforms, including intensity distributions, coefficient of variation across technical replicates, and batch effect assessment, confirming the reliability of the quantitative metabolomic dataset. **(B)** Volcano plots illustrating differentially abundant metabolites across additional pairwise comparisons beyond those presented in **Figure 4A**. Red and blue dots denote significantly upregulated and downregulated metabolites, respectively (FDR < 0.05, |log_2_FC| ≥ 1). **(C)** Extended hierarchical clustering heatmaps of metabolite subclasses (e.g., amino acids, glycerophospholipids, sphingolipids, glycolysis intermediates) across clinical groups, complementing the curated key metabolite presentation in **Figure 4C**. **(D)** Comprehensive KEGG pathway enrichment dot plots across all five clinical comparisons, with dot color encoding enrichment significance and dot size reflecting the count of enriched molecules, providingthe full pathway landscape underlying **Figure 4D**. **(E)** Additional box plots validating metabolic trends across Mild_DM, Severe_DM, and Dead_DM groups for representative metabolites beyond those highlighted in **Figure 4E**, with box bounds denoting interquartile ranges and whiskers extending to minima/maxima within 1.5× interquartile range. LC-MS, liquid chromatography-mass spectrometry; GC-MS, gas chromatography-mass spectrometry; FDR, false discovery rate; FC, fold change; KEGG, Kyoto Encyclopedia of Genes and Genomes.

Supplementary Figure 4 Supplementary multi-omics integration analyses supporting **Figure 5**. **(A)** Extended Spearman correlation heatmap of all differentially expressed proteins and metabolites across the five clinical groups, complementing the curated co-regulation modules presented in **Figure 5A**. Color intensity encodes correlation coefficients, and hierarchical clustering dendrograms delineate broad co-expression patterns. **(B)** Additional joint-pathway mapping via KEGG Mapper for core signaling axes beyond the HIF-1 pathway highlighted in **Figure 5B**, including PI3K-Akt, AGE-RAGE, and FoxO signaling. Red and blue arrows denote upregulated and downregulated molecules in the Severe_DM group, respectively, visualizing multi-omic convergence on key diabetic-severe pathways. **(C)** Topological parameter analysis of the Spearman correlation network shown in **Figure 5C**, displaying distributions of node degree, betweenness centrality, and closeness centrality to justify the selection of core regulatory hubs. **(D)** Subgroup correlation validation of top co-regulated protein-metabolite pairs in independent sample splits, confirming the robustness of the interaction modules identified in **Figures 5A–C**. KEGG, Kyoto Encyclopedia of Genes and Genomes; DM, diabetes mellitus.
