# Supplementary material for: Multi-omics reveals regulatory networks and critical early-warning factors for severe disease progression in diabetic patients infected with SARS-CoV-2
Source: Front Immunol. 2026 Sep 18;17:1871190. doi: 10.3389/fimmu.2026.1871190 (PMC13630745; doi:10.3389/fimmu.2026.1871190)
Supplement: Supplementary file 1 [file DataSheet1.docx]

**Supplementary Figures**

**Supplementary Figure S1. Muti-omics confounder adjustment analyses and quality control analysis supporting the proteomic profiling**.

(A, C) Assessment of confounding effects on protein expression and metabolites. Spearman rank correlation matrix of clinical covariates (age, BMI, sex, comorbidities, vaccine type, and dose number). All correlation coefficients clustered within the −0.5 to 0.5 range, indicating no strong inter-variable correlations or multicollinearity. (B, D) Box plot showing the variation contribution of each covariate and post-correction residual variation in omics datasets. (E) Quality control metrics for 4D-DIA mass spectrometry, including intensity distributions and coefficient of variation across replicates, confirming the reliability of the quantitative proteomic dataset. (F) Volcano plots illustrating differentially expressed proteins (DEPs) across additional pairwise comparisons beyond those presented in Figure 2B. Red and blue dots denote significantly upregulated and downregulated proteins, respectively (FDR < 0.05, |log₂FC| ≥ 1). (G) Venn diagrams depicting the overlap of DEPs among all five clinical groups, highlighting shared and group-specific molecular signatures. *Abbreviations: DEPs, differentially expressed proteins; FDR, false discovery rate; FC, fold change*.

**Supplementary Figure S2. Supplementary functional annotation analyses supporting Figure 3**. (A) GO cellular component (CC) enrichment analysis. The plot displays the cellular localization distribution of differentially expressed proteins, with bubble color encoding enrichment significance and bubble size reflecting the number of enriched molecules; key compartments include extracellular exosome and platelet alpha granule. (B) Circular chord diagram illustrating the interrelationship between key differential proteins and their enriched GO/KEGG terms. The outer ring lists functional categories, inner chords connect individual proteins to their associated terms, and chord color denotes up- (red) or down- (blue) regulation trends, visualizing the coordinated functional architecture of dysregulated proteins. (C-D) Extended KEGG pathway enrichment dot plots across additional comparisons, with dot color indicating enrichment significance and dot size reflecting the count of enriched molecules, complementing the focused pathway presentation in Figure 3B. (E) Full protein-protein interaction (PPI) network topology prior to MCODE module extraction, with node color representing differential expression direction, providing the global interaction context for the top 3 core modules highlighted in Figure 3C. (F) Sankey diagram of pathway-specific protein set analysis. The red blocks next to the gene name indicate up-regulation of the corresponding protein, blue ones exhibited down-regulation. *Abbreviations: GO, Gene Ontology; KEGG, Kyoto Encyclopedia of Genes and Genomes; PPI, protein-protein interaction; CC, cellular component; MCODE, Molecular Complex Detection*.

**Supplementary Figure S3. Supplementary metabolomic profiling analyses supporting Figure 4**. (A) Quality control metrics for integrated LC-MS and GC-MS platforms, including intensity distributions, coefficient of variation across technical replicates, and batch effect assessment, confirming the reliability of the quantitative metabolomic dataset. (B) Volcano plots illustrating differentially abundant metabolites across additional pairwise comparisons beyond those presented in Figure 4A. Red and blue dots denote significantly upregulated and downregulated metabolites, respectively (FDR < 0.05, |log₂FC| ≥ 1). (C) Extended hierarchical clustering heatmaps of metabolite subclasses (e.g., amino acids, glycerophospholipids, sphingolipids, glycolysis intermediates) across clinical groups, complementing the curated key metabolite presentation in Figure 4C. (D) Comprehensive KEGG pathway enrichment dot plots across all five clinical comparisons, with dot color encoding enrichment significance and dot size reflecting the count of enriched molecules, providing the full pathway landscape underlying Figure 4D. (E) Additional box plots validating metabolic trends across Mild_DM, Severe_DM, and Dead_DM groups for representative metabolites beyond those highlighted in Figure 4E, with box bounds denoting interquartile ranges and whiskers extending to minima/maxima within 1.5× interquartile range. *Abbreviations: LC-MS, liquid chromatography-mass spectrometry; GC-MS, gas chromatography-mass spectrometry; FDR, false discovery rate; FC, fold change; KEGG, Kyoto Encyclopedia of Genes and Genomes*.

**Supplementary Figure S4. Supplementary multi-omics integration analyses supporting Figure 5.** (A) Extended Spearman correlation heatmap of all differentially expressed proteins and metabolites across the five clinical groups, complementing the curated co-regulation modules presented in Figure 5A. Color intensity encodes correlation coefficients, and hierarchical clustering dendrograms delineate broad co-expression patterns. (B) Additional joint-pathway mapping via KEGG Mapper for core signaling axes beyond the HIF-1 pathway highlighted in Figure 5B, including PI3K-Akt, AGE-RAGE, and FoxO signaling. Red and blue arrows denote upregulated and downregulated molecules in the Severe_DM group, respectively, visualizing multi-omic convergence on key diabetic-severe pathways. (C) Topological parameter analysis of the Spearman correlation network shown in Figure 5C, displaying distributions of node degree, betweenness centrality, and closeness centrality to justify the selection of core regulatory hubs. (D) Subgroup correlation validation of top co-regulated protein-metabolite pairs in independent sample splits, confirming the robustness of the interaction modules identified in Figure 5A–C. *Abbreviations: KEGG, Kyoto Encyclopedia of Genes and Genomes; DM, diabetes mellitus.*
